# Guidelines for assessment of cardiac electrophysiology and arrhythmias in small animals

**DOI:** 10.1152/ajpheart.00439.2022

**Published:** 2022-10-21

**Authors:** Crystal M. Ripplinger, Alexey V. Glukhov, Matthew W. Kay, Bastiaan J. Boukens, Nipavan Chiamvimonvat, Brian P. Delisle, Larissa Fabritz, Thomas J. Hund, Bjorn C. Knollmann, Na Li, Katherine T. Murray, Steven Poelzing, T. Alexander Quinn, Carol Ann Remme, Stacey L. Rentschler, Robert A. Rose, Nikki G. Posnack

**Affiliations:** ^1^Department of Pharmacology, University of California Davis School of Medicine, Davis, California; ^2^Department of Medicine, Cardiovascular Medicine, University of Wisconsin-Madison School of Medicine and Public Health, Madison, Wisconsin; ^3^Department of Biomedical Engineering, The George Washington University, Washington, District of Columbia; ^4^Department Physiology, University Maastricht, Maastricht University Medical Center, Maastricht, The Netherlands; ^5^Department of Medical Biology, University of Amsterdam, Amsterdam University Medical Center, Amsterdam, The Netherlands; ^6^Department of Internal Medicine, University of California Davis School of Medicine, Davis, California; ^7^Veterans Affairs Northern California Healthcare System, Mather, California; ^8^Department of Physiology, University of Kentucky, Lexington, Kentucky; ^9^University Center of Cardiovascular Science, University Heart and Vascular Center, University Hospital Hamburg-Eppendorf with DZHK Hamburg/Kiel/Luebeck, Germany; ^10^Institute of Cardiovascular Sciences, University of Birmingham, Birmingham, United Kingdom; ^11^Department of Internal Medicine, Dorothy M. Davis Heart and Lung Research Institute, The Ohio State University, Columbus, Ohio; ^12^Department of Biomedical Engineering, Dorothy M. Davis Heart and Lung Research Institute, The Ohio State University, Columbus, Ohio; ^13^Vanderbilt Center for Arrhythmia Research and Therapeutics, Department of Medicine, Vanderbilt University Medical Center, Nashville, Tennessee; ^14^Department of Medicine, Baylor College of Medicine, Houston, Texas; ^15^Departments of Medicine and Pharmacology, Vanderbilt University School of Medicine, Nashville, Tennessee; ^16^Virginia Tech Carilon School of Medicine, Center for Heart and Reparative Medicine Research, Fralin Biomedical Research Institute at Virginia Tech, Roanoke, Virginia; ^17^Department of Biomedical Engineering and Mechanics, Virginia Polytechnic Institute and State University, Blacksburg, Virginia; ^18^Department of Physiology and Biophysics, Dalhousie University, Halifax, Nova Scotia, Canada; ^19^School of Biomedical Engineering, Dalhousie University, Halifax, Nova Scotia, Canada; ^20^Department of Experimental Cardiology, Heart Centre, Amsterdam Cardiovascular Sciences, Heart Failure and Arrhythmias Amsterdam UMC Location University of Amsterdam, Amsterdam, The Netherlands; ^21^Cardiovascular Division, Department of Medicine, Washington University in Saint Louis, School of Medicine, Saint Louis, Missouri; ^22^Department of Cardiac Sciences, Libin Cardiovascular Institute, Cumming School of Medicine, University of Calgary, Calgary, Alberta, Canada; ^23^Department of Physiology and Pharmacology, Libin Cardiovascular Institute, Cumming School of Medicine, University of Calgary, Calgary, Alberta, Canada; ^24^Sheikh Zayed Institute for Pediatric Surgical Innovation, Children’s National Hospital, Washington, District of Columbia; ^25^Department of Pediatrics, George Washington University School of Medicine, Washington, District of Columbia

**Keywords:** arrhythmia, cardiac electrophysiology, ECG, guidelines, small animals

## Abstract

Cardiac arrhythmias are a major cause of morbidity and mortality worldwide. Although recent advances in cell-based models, including human-induced pluripotent stem cell-derived cardiomyocytes (iPSC-CM), are contributing to our understanding of electrophysiology and arrhythmia mechanisms, preclinical animal studies of cardiovascular disease remain a mainstay. Over the past several decades, animal models of cardiovascular disease have advanced our understanding of pathological remodeling, arrhythmia mechanisms, and drug effects and have led to major improvements in pacing and defibrillation therapies. There exist a variety of methodological approaches for the assessment of cardiac electrophysiology and a plethora of parameters may be assessed with each approach. This guidelines article will provide an overview of the strengths and limitations of several common techniques used to assess electrophysiology and arrhythmia mechanisms at the whole animal, whole heart, and tissue level with a focus on small animal models. We also define key electrophysiological parameters that should be assessed, along with their physiological underpinnings, and the best methods with which to assess these parameters.

## INTRODUCTION

Cardiac arrhythmias are a major global health burden and a primary cause of morbidity and mortality in a wide spectrum of patients, particularly those with existing cardiovascular disease, including atherosclerosis, coronary heart disease, heart failure, and myocardial infarction (1, 2). Cardiac arrhythmias occur in 1% of all individuals aged <55 yr and in up to 5% of those aged >65 yr (3), with a total direct annual healthcare cost summing up to $67 billion in the United States (4). In particular, sudden cardiac death (SCD) secondary to cardiac arrhythmias is a leading cause of mortality in the Western world, accounting for up to 20% of all natural deaths and up to 50% of all cardiovascular deaths (5). Cardiac arrhythmias, therefore, constitute an increasing global health burden, and their prevalence is increasing as the population ages. Thus, the development of novel efficient preventive and therapeutic strategies is essential, which requires in-depth insight into disease mechanisms underlying both atrial and ventricular arrhythmias. Preclinical animal studies of cardiovascular disease are a mainstay for understanding pathological remodeling, drug development and testing, as well as for optimizing pacing (6, 7) and defibrillation strategies (8, 9). Although human-induced pluripotent stem cell-derived cardiomyocytes (iPSC-CM) and iPSC-CM-based two-dimensional (2-D)/three-dimensional (3-D) constructs allow for drug screening or testing patient-specific genetic mutations in human cells (10–12), iPSC-CM platforms have a number of drawbacks limiting their use to study cardiac arrhythmias. These include a different cellular electrophysiological phenotype compared with mature adult cardiomyocytes (13, 14), lack of a heterocellular tissue composition [cardiomyocytes represent only ∼50 and ∼30% of cardiac cells by cell number in ventricles and atria, respectively (15)], anisotropic fiber orientation (16, 17), heart chamber specificity (atria, ventricle, sinus node), and 3-D organization (18). Therefore, small and large animal models remain essential for the study of cardiac electrophysiology and arrhythmia mechanisms.

These guidelines will examine experimental approaches for the assessment of cardiac electrophysiology and arrhythmia mechanisms at the whole animal, whole heart, and tissue level, with a focus on small animal models. Although experiments in isolated cardiomyocytes or cellular-based tissue constructs are vitally important and often necessary to discern underlying cellular mechanisms, we will not cover those approaches here and refer readers to excellent reviews on these topics (19–22). The purpose of these guidelines is to *1*) provide an overview of experimental techniques commonly used for electrophysiological assessments in small animal models, *2*) define key electrophysiological measurements and their physiological underpinnings, *3*) provide recommendations and key considerations that researchers should take into account when designing studies and using these experimental techniques, and *4*) provide an extensive reference list that may serve as a useful guide for those interested in implementing experimental electrophysiological approaches in animal models.

## EVALUATION OF CURRENT LITERATURE

To obtain insight into current methods and approaches that are commonly used for measuring cardiac electrophysiology and arrhythmias in animal models, we evaluated recently published articles in the *American Journal of Physiology-Heart and Circulatory Physiology*. The search included all primary research articles published from January 1, 2019 to May 3, 2022 using the terms “electrophysiology” or “arrhythmia.” This search identified 82 articles that were then evaluated by two authors (C.M.R. and N.G.P.) to only include original research articles that reported one or more electrophysiological measurements from animal models obtained from the intact heart, intact tissue, or in vivo. Articles using only human tissues or cell-based work (primary isolated cardiomyocytes or iPSC-derived cardiomyocytes) were excluded, resulting in 30 articles for further evaluation.

**Figure 1.**
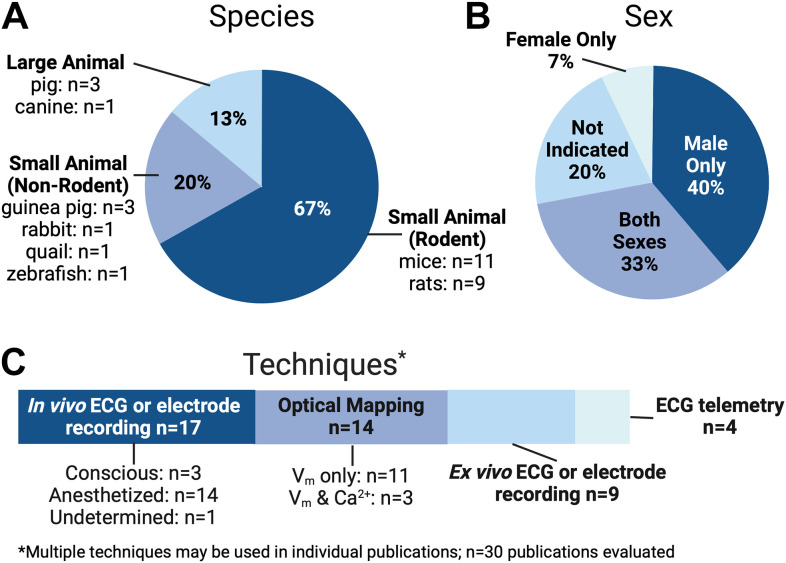
Evaluation of recent literature published in *Am J Physiol Heart Circ Physiol* (2019–2022). Breakdown of published studies according to species studied (*A*), animal sex in each study (*B*), and major techniques used (*C*). Many of these experimental studies combined multiple techniques, including in vivo and ex vivo approaches, for comprehensive characterization. *n* = 30 publications evaluated. Created by Biorender.com and published with permission.

A summary of our search results is shown in Fig. 1. In terms of species used, the majority of these studies (∼67%) used rodents, with approximately equal distribution between mice (23–33) and rats (34–42). Guinea pig (43–45) and porcine hearts (46–48) were each used in ∼10% of studies, followed by a small number of reports in rabbit (49), zebrafish (50), quail (51), and canine (52) hearts. Overall, there was good reporting of sex, age, and strain, yet only ∼30% of studies evaluated both male and female animals, whereas ∼40% exclusively reported data from male animals. When we consider the known sex differences in electrophysiological properties and arrhythmia susceptibility (53), this is an area where improvement in experimental design and consideration of sex as a biological variable could have important physiological implications.

In terms of disease models, genetically modified mice (23, 26, 28–33) or specific rat strains (e.g., spontaneously hypertensive rat) (34, 35, 40) were most commonly assessed (∼37% of studies), and myocardial infarction, ischemia, or hypoxia were evaluated in ∼27% of studies (25, 31, 35, 38, 42, 44, 45, 47). Around 16% of investigations used pharmacological modulation (24, 43, 49, 50, 52). A small number of studies investigated environmental effects [including herbicide (36), vaping (27), and alcohol (51)], aging and development (25, 28, 41), nonischemic heart failure (39), and the remaining studies performed electrophysiological assessments in nondiseased hearts (37, 46, 48). The most commonly used techniques to evaluate electrophysiological parameters and arrhythmia susceptibility were in vivo electrocardiogram (ECG) or electrode recordings in conscious or anesthetized animals (∼56% of studies) (23–28, 30, 32, 36, 38, 40–42, 46–48, 52) or optical mapping in excised hearts or tissues (∼47% of studies) (27, 30, 31, 33–35, 39, 41, 43–45, 49–51). The remaining studies used electrode-based or ECG recordings in excised hearts and tissues (24, 25, 29, 31, 32, 35, 40, 41, 50) or in vivo ECG telemetry (27, 29, 33, 37), and often, a combination of multiple approaches were used to rigorously evaluate electrophysiological properties (including experiments in isolated cardiomyocytes, which are not covered in detail in these guidelines).

Considering the heavy reliance on small animals for many recent cardiac electrophysiology studies, these guidelines will focus on best practices for experimental approaches most often used in small animals (e.g., mice, rats, guinea pigs, and rabbits). However, some of the measurements, experimental techniques, and recommendations can be universally applied to a variety of species.

## CHOICE OF ANIMAL MODEL

**Figure 2.**
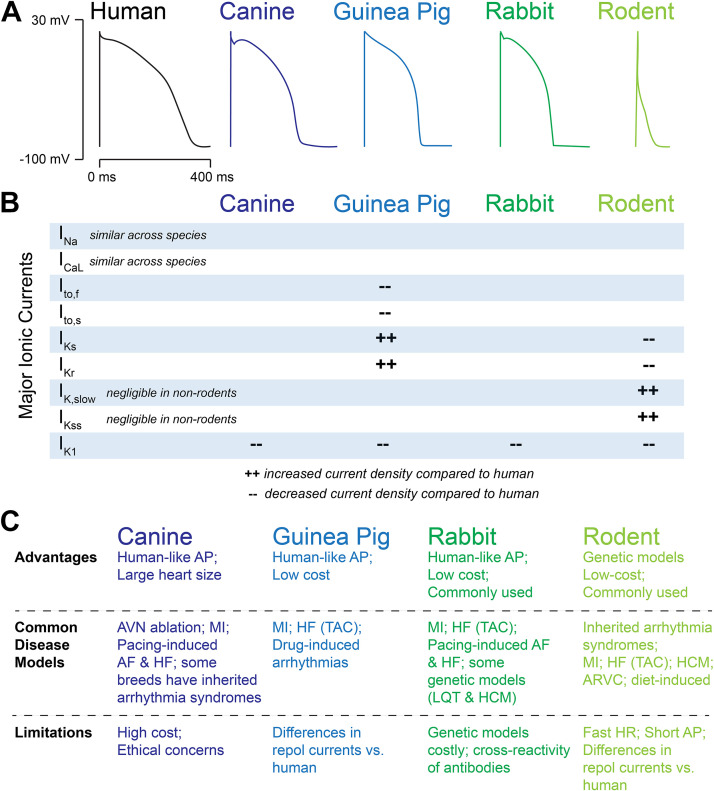
*A*: schematic showing approximate ventricular action potential shapes/durations between species. *B*: major differences in underlying ionic currents compared with the human action potential (AP). *C*: brief list of advantages, commonly used disease models, and limitations for each species. AF, atrial fibrillation; ARVC, arrhythmogenic right ventricular cardiomyopathy; AVN, atrioventricular node; HCM, hypertrophic cardiomyopathy; HF, heart failure; HR, heart rate; LQT, long QT syndrome; MI, myocardial infarction; TAC, transverse aortic constriction. Partially adapted and redrawn from Refs. 20 and 56 with permission.

Although these guidelines focus on experimental techniques for small animals, appropriate choice of an animal model is highly dependent on the research question and type of arrhythmia to be investigated. For some studies, in vivo and ex vivo assessments could be combined with in vitro molecular analyses, measurements from isolated cardiomyocytes, and computational modeling. Important considerations include anatomical features, such as heart size and macrostructure, as well as species-dependent differences in heart rate (HR), ionic currents, and autonomic regulation (54, 55). For example, while the fast upstroke of the action potential (AP) (mediated by the sodium current Na_v_1.5) is largely preserved across species, the plateau and repolarization phases of the ventricular AP differ significantly across species (Fig. 2). Dogs, pigs, guinea pigs, and rabbits have ventricular AP characteristics largely similar to humans, although guinea pigs, pigs, and rabbits are somewhat limited by distinctive differences in the transient outward current *I*_to_. Many of the K^+^ currents underlying cardiac repolarization in mice and rats are also different from humans (7, 20, 57–60). Significant chamber and regional differences exist within and between species (61, 62), as does arrhythmia susceptibility. For example, in large animals, pigs are more susceptible to ventricular arrhythmias and sudden cardiac death (63, 64), whereas goats are a preferred model for atrial fibrillation (60, 65). Although large animals have been used to study electrophysiological changes and arrhythmogenesis during pathological conditions such as myocardial ischemia, infarction, and heart failure (66–68), in many instances, small animals are preferred because of the availability of transgenic species, shorter life spans, and ease of use, which complements studies of aging, chronic disease, studies with multiple experimental groups, and focused hypotheses that can be tested using transgenic animals (25, 60, 69, 70).

For genetic studies, neonatal cardiomyocytes from rats, mice, or rabbits allow for overexpression or knockdown of genes followed by electrophysiological assessment, but their immaturity in ion channel isoform expression and t*-*tubule structure is an important limitation. These limitations can be partly overcome using genetically altered mice, rats, or rabbits (71, 72). Mice are easy to breed and genetically modify in vivo through overexpression, deletion, or mutation of genes of interest. Rodent studies also allow for investigation of the impact of the genetic background using distinct inbred strains (73). More recently, rabbits have been used in transgenic studies, which are of particular value when investigating K^+^ channel genes and mutations (72). Overall, transgenic animals allow for in-depth electrophysiological studies at the in vivo, whole heart, and cardiomyocyte levels, combined with histological and molecular analyses, as well as potential therapeutic investigations. For a detailed discussion of mammalian models used for various electrophysiological studies, we refer to excellent recent reviews and position papers on this topic (7, 20, 59, 60).

Alternative animal models that are easier for gene manipulation in electrophysiological studies include *Drosophila melanogaster* and zebrafish. Although zebrafish have distinct morphological differences in cardiac anatomy compared with humans, their functional similarities (e.g., AP morphology), high conservation of gene function, simplified maintenance, short life span, and straightforward genetic manipulation (74–76) make them attractive for high-throughput screening of gene function, as well as druggable targets, which can be further validated in mammalian models. In summary, each animal model has important advantages and disadvantages that must be considered when designing an electrophysiological study, including strain-dependent anatomical and biophysical differences, along with practical and ethical considerations.

## QUANTIFYING ELECTROPHYSIOLOGY

Before introducing specific experimental techniques used to assess whole animal, whole heart, and tissue-level electrophysiology, we provide a comprehensive overview of recommended electrophysiological parameters and the physiological basis of such parameters. It should be noted that many of these electrophysiology end points are technique independent, meaning that they can be assessed using different experimental approaches and the detailed analysis protocols may differ for each technique, but the underlying (electro)physiological foundations are the same.

It is also worth noting that there are myriad end points that may be assessed to discern underlying mechanisms in both healthy and diseased hearts. Therefore, there is no standard recommendation for which parameters should be measured to characterize a specific disease model. Electrophysiological assessments should be guided a priori by a clear hypothesis. For example, when factors that may influence activation or wave propagation are suspected to be at play (e.g., Na^+^ channel function, cell-cell coupling, structural remodeling), measurements of QRS duration, AP upstroke, activation patterns, conduction velocity, and/or anisotropy should be assessed. On the other hand, if repolarizing factors are suspected, measurements of QT interval, action potential duration (APD), refractory periods, or repolarization heterogeneity should be quantified. Within each subsection below, the citations include many exemplary studies in which these parameters have been used to characterize various disease models. The section *Arrhythmogenesis* further explains how alternations to specific endpoints may be pro- or antiarrhythmic.

### Characterization of the Sinus Node

**Figure 3.**
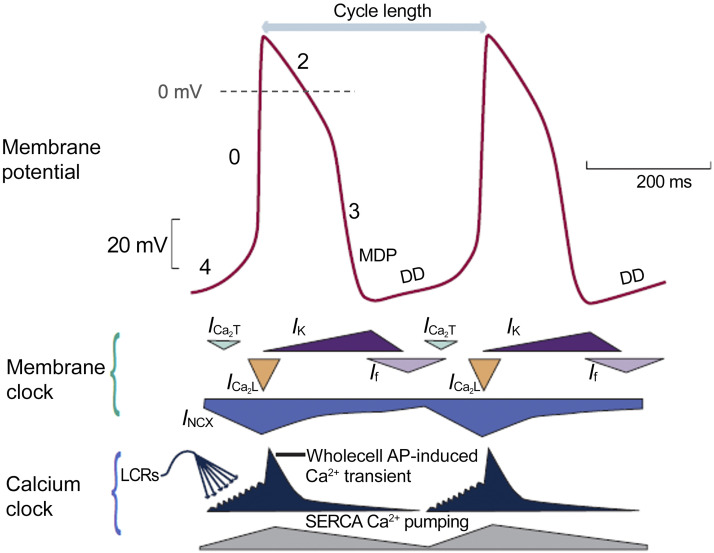
Schematic of sinus node membrane potential and underlying mechanisms. *Top*: action potential (AP) of a rabbit sinoatrial nodal myocyte (red). *Middle*: different components of the “membrane clock.” *Bottom*: different components of the “Ca^2+^ clock.” During phase 4, cytosolic [Ca^2+^] increases due to spontaneous Ca^2+^ releases from the sarcoplasmic reticulum (SR) (dark blue). Activation of L-type Ca^2+^ channels (orange) then causes Ca^2+^-induced Ca^2+^ release from the SR, resulting in the whole cell Ca^2+^ transient. Cytoplasmic Ca^2+^ is removed by the sarco(endo)plasmic reticulum calcium ATPase (SERCA) pump (gray) and by sodium-calcium exchanger (NCX) (blue). DD, diastolic depolarization; *I*_Ca,T_, T-type voltage-dependent Ca^2+^ current; *I*_Ca2,L_, L-type voltage-dependent Ca^2+^ current; *I*_f_, funny current; *I*_NCX_, sodium-calcium exchange current; *I*_K_, delayed rectifier potassium current; LCRs, local Ca^2+^ releases; MDP, maximum diastolic potential. Reproduced from Ref. 79 with permission.

The spontaneous beating of sinoatrial nodal myocytes is initiated, sustained, and regulated by a dynamic interaction between electrogenic membrane proteins [the “voltage (*V*_m_) clock”] and intracellular Ca^2+^ cycling (“the Ca^2+^ clock”) (77–79). Pacemaker cells are characterized by the presence of spontaneous diastolic depolarization, including an early and late phase (80) (Fig. 3). The components of the *V*_m_ clock, *I*_f_, *I*_Ca,T_, and *I*_Ca,L_ [particularly due to Ca_V_1.3 channels (81)] contribute to early diastolic depolarization. Then, spontaneous local Ca^2+^ release events from the sarcoplasmic reticulum (SR) via ryanodine receptors (RyR) generate small increments in intracellular Ca^2+^ concentration. These local calcium release events activate the Na^+^/Ca^2+^ exchanger (NCX), which generates an inward *I*_NCX_ current and boosts the diastolic depolarization rate, resulting in the onset of an AP via activation of *I*_Ca,L_. The function of *I*_f_, *I*_Ca,T_, and *I*_Ca,L_ currents can be indirectly estimated from the early (slow) component of diastolic depolarization, whereas the late (and faster) diastolic depolarization component can be used to assess NCX function, as well as the contribution of local Ca^2+^ release events. Diastolic depolarization can be measured via both electrode-based and optical mapping techniques, but the measurement of local Ca^2+^ release events requires optical techniques that use Ca^2+^ sensitive indicators (82).

Several key parameters should be measured to characterize sinus node function in vivo: *1*) spontaneous heart rate (HR) assessed at baseline and in response to autonomic stimulation and/or blockade; *2*) HR variability [HRV: beat-to-beat variation in cycle length, from which several time and frequency domain metrics, and other nonlinear metrics can be analyzed (83)] to characterize neurohormonal regulation and sinus node function (84–86); *3*) incidence and duration of sinus node pause to characterize the stability of sinus node pacemaking; and *4*) sinus node recovery time (SNRT) to determine the magnitude of the funny current, calcium clock, and activity of the Na^+^/K^+^ ATPase. SNRT can also be used to evaluate sinus node dysfunction and detect sick sinus syndrome. SNRT is measured after the suppression of spontaneous sinus node beating by fast atrial pacing, is equal to the time interval between the last pacing beat and the first spontaneous beat, and can be corrected (cSNRT) by subtracting the baseline resting cycle length, which corrects for HR and allows for comparison between animals.

Sinus node function can also be studied by optical mapping (either in isolated sinus node preparations or from the posterior epicardial surface of the intact atria/heart) and the following additional parameters may be assessed: *1*) sinoatrial delay during spontaneous rhythm, measured as the time between the earliest excitation within the sinus node and first atrial activation [this can be used to characterize electrical conduction within the sinus node and through the specialized conduction pathways (87)]; *2*) localization of the leading pacemaker and pacemaker shift that can occur in a beat-to-beat manner in response to neurohormonal or mechanical stimulations, or during pathophysiological conditions (88, 89); and *3*) localization and distribution of subsidiary atrial pacemakers, which may also be identified by P-wave morphology (i.e., fractionation, inversion) or duration of the PQ interval on the ECG.

### Characterization of the Atrioventricular Node

The intramural location of the atrioventricular node (AVN) at the posteroinferior region of the interatrial septum (requiring atrial dissection) makes it difficult to use electrode-based techniques to directly record AVN activation. Accordingly, AVN function is frequently assessed via ECG by determining its conduction at increasingly rapid rates and its refractory properties with progressively premature stimulation in the right atrium (90, 91). Wenckebach periodicity is a measurement of AVN refractoriness and is assessed with increasingly rapid dynamic atrial pacing (S1-S1, decreasing cycle length) until ventricular conduction fails. A similar approach is used to identify 2:1 atrioventricular (AV) conduction block by determining the longest S1-S1 coupling interval that results in 2:1 block. The AVN effective refractory period (AVNERP) is the shortest S1-S2 interval that still leads to ventricular activation (41, 91). This can be determined by reducing the S1-S2 interval until capture is lost or by increasing the S1-S2 interval until capture. The last approach will lead to a longer refractory period because of short-term cardiac memory.

The AV delay has been associated with progressively decreasing AP amplitude and loss in maximal diastolic *V*_m_ because of incomplete repolarization during rapid atrial rhythm (92, 93). A reduced safety factor, i.e., the available depolarizing current (94), results in progressively longer beat-to-beat AVN conduction times and, ultimately, in AVN block at the Wenckebach cycle length. The pause created with the blocked AVN conduction is associated with the restoration of maximal diastolic *V*_m_ and reinitiation of the conduction cycle via the AVN. Wenckebach periodicity may be linked to the presence of fast and slow conduction pathways within the AVN, each with unique electrophysiological properties (92, 95). The presence of at least two atrionodal pathways supporting the induction of reentry has been demonstrated in rabbits (95–98), but animal models of spontaneous AVN reentrant tachycardia have not been described. Finally, the AVN can display automaticity and function as an independent pacemaker in the setting of suppressed sinus node function, either during pathological conditions or under strong parasympathetic stimulation. AVN junctional rhythms are linked to latent pacemakers within the AVN (99) and are significantly slower than normal sinus rhythm.

### Atrial and Ventricular Parameters

#### Assessment of atrial and ventricular activation and repolarization.

Atrial and ventricular activation and repolarization during spontaneous sinus rhythm represent the normal pattern of electrophysiological activity. This can be characterized by atrial and ventricular activation time, repolarization time, as well as AP and, if optical methods are used, Ca^2+^ transient (CaT) morphology and parameters (including relationships between the AP and CaT as measures of excitation-contraction coupling, see *Ca^2+^ transient characteristics*). These parameters can be correlated with the corresponding ECG parameters, including P wave and QRS duration for atrial and ventricular activation, respectively, and the T wave for ventricular repolarization. Because AP and CaT properties and propagation depend heavily on HR (i.e., restitution), these parameters should be measured during constant electrical pacing or potentially corrected for HR (e.g., QTc). Although, HR correction may not be required in mice, as the QT interval is not significantly altered by HR, unless the R-R interval becomes significantly shortened (100–102).

##### Action potential characteristics.

Although transmembrane potential (*V*_m_) measured using sharp glass micropipette electrodes represents a gold standard for the analysis of AP characteristics, many AP characteristics can be measured using other electrode-based or optical mapping techniques. For method-specific electrophysiological measurements, see experimental approaches.

**Figure 4.**
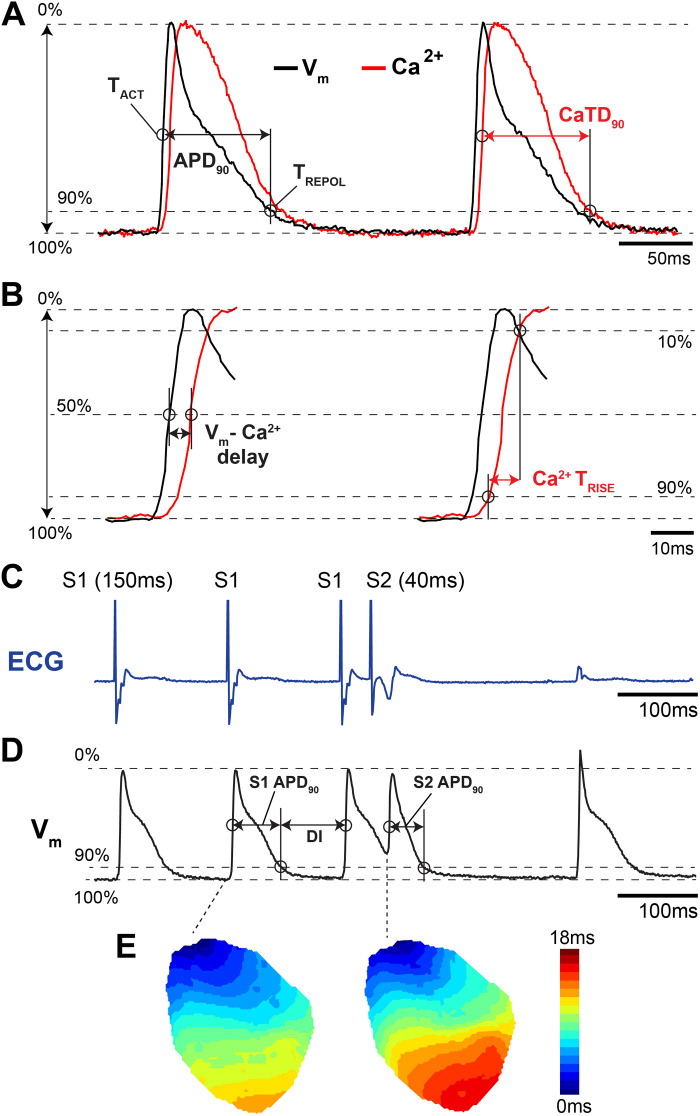
Example action potential (AP) and calcium transient (CaT) measurements from an isolated mouse heart. *A* and *B*: optical recording of *V*_m_ and Ca^2+^ illustrating several common measurements. *T*_ACT_, activation time; *T*_REPOL_, repolarization time; *T*_RISE_, rise time. *C*: electrocardiogram (ECG) recording during an S1-S2 pacing protocol. Vertical spike on the ECG represents ventricular pacing artifact. *D*: corresponding optical action potentials during S1-S2 pacing protocol. DI, diastolic interval. *E*: representative activation maps illustrating slower total conduction during the S2 stimulus. Adapted from Refs. 103 and 104 with permission.

Important AP characteristics that are typically measured include resting *V*_m_, activation time, AP amplitude, AP overshoot, upstroke velocity, repolarization time, and AP duration (APD). Activation time can be measured as the time of maximum derivative during *phase 0* (upstroke velocity, d*V*/d*t*_max_), or alternatively, as the time at which the AP reaches 50% of its maximal amplitude (Fig. 4, *A* and *B*). Repolarization time can be measured as the time of the maximum second derivative after the upstroke, which corresponds to the inflection point as the AP transitions from repolarization (*phase 3*) to rest (*phase 4*). Notably, the second derivative is highly sensitive to noise and postprocessing filtering. Therefore, repolarization time is often measured as the time at which the AP has repolarized from its peak by a percentage of the AP amplitude, e.g., repolarized by an amount equal to 30, 50, or 90% of the AP amplitude. APD is measured as the difference between repolarization and activation time (Fig. 4*A*). Many of these AP characteristics may be used to indirectly estimate the contribution of ionic currents to the AP. For example, resting *V*_m_ (*phase 4*) mainly reflects the function of the inwardly rectifying K^+^ current (*I*_K1_), the Na^+^/K^+^-ATPase, and other background currents. AP amplitude and upstroke velocity (d*V*_m_/d*t*) reflect the function of fast Na^+^ channels. APD can be used to indirectly assess the contribution of other ionic currents, including transient outward K^+^ current *I*_to_ (at APD_20–30%_), *I*_Ca,L_ and *I*_NCX_ (at APD_50%_), *I*_Ks_, *I*_Kr_, *I*_Kur_, and *I*_K1_ (at APD_90%_), although the relative contribution of these currents throughout the AP are species and location specific (7, 20, 105). These AP measurements cannot precisely discern individual current amplitudes and kinetics, which requires patch clamping of isolated myocytes, but may be useful for general assessment of the balance between inward and outward currents.

##### Activation mapping.

When multidimensional electrode array or optical approaches are used to map electrical activity, the AP activation time measured at each electrode or pixel can be used to reconstruct the location and speed of propagating wavefronts. It is common to visualize propagation sequences as an isochronal map (iso = same; chronal = time). These often-colorful maps have lines or colored bands that indicate areas of tissue that depolarized within the same time interval (Fig. 4*E*). A valuable feature of such maps is that crowding of the isochrones signifies conduction slowing while the spreading of isochrones signifies fast conduction (106). In this way, isochronal maps capture the complex spatiotemporal dynamics of propagation sequences as a single two-dimensional image, providing clear visualization of slow and fast conduction, conduction block, and reentrant pathways (107).

##### Conduction velocity.

**Figure 5.**
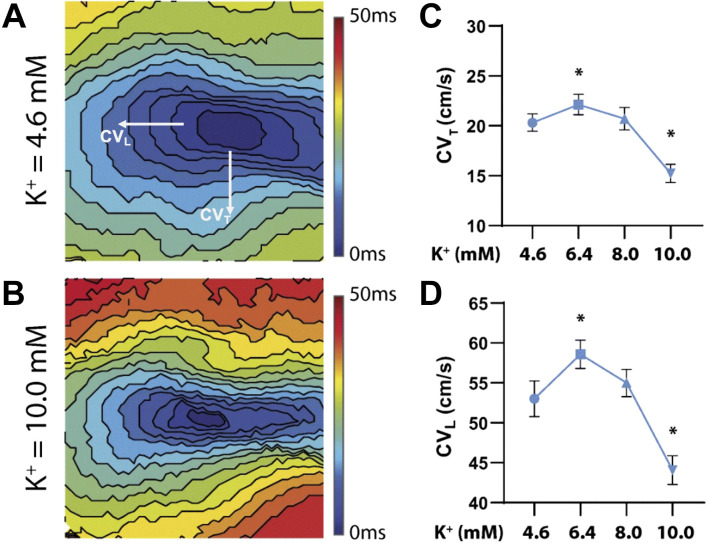
Conduction velocity measurements. *A* and *B*: epicardial isochronal maps generated from optically mapped guinea pig hearts perfused with increasing potassium concentrations. Transverse (CV_T_) and longitudinal (CV_L_) conduction velocity designated for visualization purposes. *C* and *D*: summary of CV_T_ (*C*) and CV_L_ (*D*) as a function of potassium concentration. Adapted from Ref. 115 with permission. *P < 0.05, significantly different compared with 4.6 mM K^+^.

Atrial and ventricular conduction velocity (CV) is spatially heterogeneous and depends on multiple factors, including cardiomyocyte size, fiber orientation, the expression level, and spatial distribution of gap junctions and Na^+^ channels, the distribution of collagen, and the pattern of excitation (108–112). CV as a tissue property is ideally measured during electrical pacing to control the initiation of wavefronts and the cycle length, as CV slows at short coupling intervals (see Fig. 4*E* and *Restitution*). CV should be measured along the fastest and slowest directions, which typically align with longitudinal and transverse fiber orientations, respectively (113, 114) (Fig. 5). Ventricular CV is usually not measured during sinus rhythm or endocardial pacing because propagation through the specialized conduction system causes wavefront breakthrough patterns that introduce CV measurement errors. Conduction anisotropy, typically between two and three for normal cardiac tissue, is an important tissue parameter that corresponds to the ratio between longitudinal and transverse CV (116). Notably, increased values of conduction anisotropy may be proarrhythmic and promote reentry (109). With two electrodes placed at different locations on the tissue, CV can be computed as the distance between the electrodes divided by the difference in activation time measured at each electrode. However, this assumes that the wavefront travels along a straight line connecting the electrodes. If the wavefront travels at an angle or perpendicular to the line connecting the two electrodes, a nearly identical activation time would then be measured at each electrode, giving rise to artificially high CV values. Mapping of activation sequences using electrode arrays or optical mapping overcomes this limitation, whereby spatiotemporal fitting algorithms are used to calculate conduction speed and direction throughout the mapped region (117–119). There are multiple variations of the basic CV measurement described here so detailed reporting of the specific measurement approach used in an experiment is necessary to compare results across laboratories (120).

##### APD and repolarization mapping.

The expression pattern (105) and biophysical properties of proteins associated with transmembrane currents are often chamber specific (121) and heterogeneously distributed within regions of the heart (e.g., transmural and apicobasal gradients). This heterogeneity may be assessed by measuring APD at many sites and then quantifying the difference or dispersion in APD between those sites. The standard deviation of the APD, or the interquartile range of APD, across a region can be used as a measure of APD dispersion (see experimental approaches). APD and repolarization maps, reconstructed using high-resolution mapping, can be used to identify areas with increased heterogeneity of tissue repolarization, which is a primary cause of reentrant activity. However, such maps do not reveal postrepolarization refractoriness, which may occur during ischemia, wherein tissue repolarization can occur before recovery from refractoriness (122). In this setting, dispersion of repolarization would not be the best assessment of the arrhythmogenic substrate. Both the AP morphology and APD of electrically coupled cardiomyocytes are modulated by electrotonic load, which homogenizes differences in APD between cells. Therefore, APD maps do not completely reveal the expression heterogeneity of proteins associated with the cardiomyocyte AP (123, 124).

##### Restitution.

**Figure 6.**
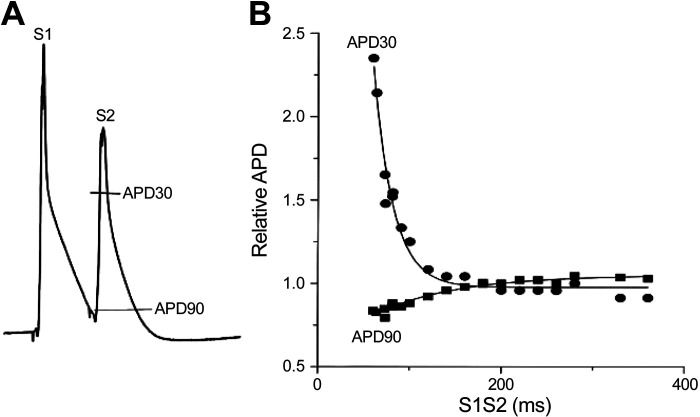
Action potential duration (APD) restitution in the mouse heart. *A*: monophasic action potentials recorded from an isolated mouse heart during an S1-S2 pacing protocol. *B*: example restitution curves of early (APD_30_) and late (APD_90_) repolarization. Reprinted from Ref. 126 with permission.

Electrophysiological adaptation to heart rate is called restitution and describes how electrophysiological parameters (e.g., APD, CaT, and CV) depend on the preceding diastolic interval (the rest period between repolarization and the next depolarization). In large nonrodent mammals, APD shortens with reductions in the diastolic interval because a significant fraction of K^+^ channels (mainly those carrying *I*_Ks_) activated during the previous beat remain open, resulting in greater peak K^+^ current than at longer diastolic intervals (125). Rodent hearts lack *I*_Ks_, and late repolarization is driven in part by the Na^+^/Ca^2+^ exchanger (*I*_NCX_). APD restitution (at the time of APD_90_) in the mouse heart is also dependent on the recovery of SR Ca^2+^ release at short diastolic intervals (126). Early repolarization (at the time of APD_30_) in rodent hearts has been shown to prolong, rather than shorten, at short diastolic intervals because of incomplete recovery of *I*_to_, which is the main driver of early repolarization in rodents (126) (Fig. 6). Restitution of CV occurs at short diastolic intervals when the time of electrical excitation encroaches upon the tissue refractory period when Na^+^ channels have not fully recovered from inactivation, leading to reduced excitability.

Rate-related changes in APD, CaT, and CV are implicated in both normal physiology and arrhythmogenesis (127), as further discussed in *Arrhythmogenesis*. Restitution parameters should be determined using either standard or dynamic restitution pacing protocols (128). The standard restitution protocol uses an S1-S2 pacing protocol, where a train of basic pacing pulses (S1, typically ∼20) is delivered followed by a premature stimulus (S2) at progressively decreasing coupling intervals between the last S1 and the S2, until excitation by the premature stimulus (S2) is blocked (Fig. 4, *C* and *D*). The APD of the S2 beat (and/or the corresponding CV or CaT duration/amplitude) is measured and plotted as a function of the preceding diastolic interval, which is equal to the S1-S2 interval minus APD of the response to the last S1 stimulus.

For the dynamic restitution protocol, the myocardium is paced at a constant pacing cycle length close to the normal sinus rhythm cycle length. After ∼50 stimuli (or ∼30 s of pacing) at this pacing cycle length, the pacing is stopped and APD, CV, or CaT duration and/or amplitude of the last paced beat is measured. Pacing is then reinitiated at a progressively shorter cycle length and measurements continue until either 2:1 block or higher-order periodicities occur. Similar to the standard restitution protocol, for each pacing cycle length, the APD, CV, or CaT duration/amplitude are plotted as a function of the preceding diastolic interval.

##### Refractory period.

The longest S1-S2 interval for the standard restitution protocol that fails to elicit a propagated response is the effective refractory period. Similarly, the shortest pacing interval to capture without block during the dynamic protocol is the functional refractory period. Both effective and functional refractory periods are heavily dependent on the length of inactivation time of fast Na^+^ channels. Depending on the pacing location, protocols can be implemented to identify atrial, AVN, and ventricular effective refractory periods (i.e., AERP, AVNERP, and VERP) (90, 91, 129).

##### Ca^2+^ transient characteristics.

For CaT morphology, Ca^2+^-sensitive fluorescent indicators must be used along with optical recording methods (see *Recording of the optical Ca^2+^ transient* for a discussion of Ca^2+^-sensitive indicators and recording methods). CaT rise time (typically calculated as the time it takes for the upstroke to go from 10 to 90% of the amplitude, Fig. 4*B*) can provide an assessment of Ca^2+^-release synchronization (130) and is often reported as a correlate of Ca^2+^-induced Ca^2+^-release kinetics in much the same way that the upstroke of contraction or intraventricular pressure measurement describes contractility (inotropy). The CaT amplitude is representative of a combination of *I*_Ca,L_ and SR Ca^2+^ release, which varies by species with ∼70% of total intracellular Ca^2+^ increase coming from the SR in rabbits and larger mammals and ∼90% coming from the SR in rodents (with *I*_Ca,L_ contributing the remaining portion) (131). The decay of the CaT is an index of Ca^2+^ resequestration (by the SR Ca^2+^ ATPase, SERCA pump) and Ca^2+^ efflux from cardiomyocytes (by *I*_NCX_). The relative contribution of SERCA versus *I*_NCX_ in intracellular Ca^2+^ decay is species dependent and, at steady state, matches the proportions of Ca^2+^ released from the SR and Ca^2+^ carried into the cell via *I*_Ca,L_, respectively (131). Rate of CaT decay is often quantified by either the duration of the CaT at 50% return to baseline or as the decay time constant or τ of an exponential fit. Region-specific distribution of CaT parameters may also be measured to assess the variability of Ca^2+^ handling.

Several parameters related to excitation-contraction coupling can be investigated when optical APs are recorded concurrently with CaTs. For example, to assess the coupling between L-type Ca^2+^ channels and RyRs, the AP-to-CaT delay can be measured (time between AP activation and CaT activation at each location, Fig. 4*B*) (132). Moreover, phase plots of the *V*_m_/Ca^2+^ relationship can be constructed by plotting the normalized fluorescent *V*_m_ values on the *x*-axis against the normalized fluorescent Ca^2+^ values on the *y*-axis over the time course of a single beat. The chirality of these plots indicates either normal *V*_m_-Ca^2+^ coupling (counterclockwise, *V*_m_ upstroke precedes Ca^2+^ rise) or abnormal Ca^2+^-mediated triggered activity (clockwise, Ca^2+^ release precedes *V*_m_ depolarization) (132, 133). Finally, the difference between the CaT duration and APD at each location can be used to assess the relationship between Ca^2+^ reuptake/extrusion and repolarization, where significantly elevated intracellular Ca^2+^ after repolarization represents a vulnerable time for Ca^2+^-mediated reexcitation (134).

#### Arrhythmogenesis.

Although technical and methodological details on arrhythmia testing protocols are summarized in Recording of the optical action potential, here we provide a brief theoretical foundation behind commonly used protocols for arrhythmia induction. It should be noted that the exact electrophysiological mechanisms of cardiac arrhythmias are not completely understood and remain under intensive investigation. In general, cardiac arrhythmias could be classified into categories of disorders of impulse formation (such as enhanced and abnormal automaticity as well as triggered activity), disorders of impulse conduction (such as block of conduction, with or without reentry), or a combination of both. Because of distinct underlying cellular and molecular mechanisms, different types of arrhythmias require different provocative protocols. For example, for arrhythmias that involve abnormal conduction, fast-pacing protocols (S1-S1) or premature stimuli (S1-S2) are commonly used to assess arrhythmia susceptibility. The initiation of such arrhythmias is associated with the development of local conduction discontinuities and is best described by restitution theory. In contrast, disorders of impulse formation may stem from abnormal automaticity or Ca^2+^-dependent triggered activity (e.g., early or delayed afterdepolarizations, EADs, DADs). At slow heart rates, hearts are more susceptible to ectopic activity (due to the longer diastolic interval that allows the escape of this activity) or EAD-based triggered activity, which is promoted by the long APD that occurs at slow heart rates. Posttachycardia pauses tend to promote DAD-based triggered activity (due to loading of the SR with Ca^2+^ at high heart rates). In pathological conditions, sinus node dysfunction may therefore also play a role in susceptibility to triggered activity, but experimentally, pacing protocols that mimic these rate-dependent phenomena should be used to provoke the suspected arrhythmia mechanism. In some cases, spontaneous arrhythmias that involve abnormal impulse formation may also require pharmacological “stress tests” as discussed in Recording of the optical action potential.

##### Slope of restitution and cardiac alternans.

The restitution properties of myocardial tissue have been linked to its arrhythmogenic potential (135, 136). The restitution hypothesis states that the slope of the APD restitution curve is the main determinant of wave break (137), with steeper slopes associated with increased susceptibility to reentrant arrhythmias. However, some experimental studies have found that the precise value of the APD restitution slope may not be a strong predictor of arrhythmogenesis (138, 139). According to theory, when the slope of the APD restitution curve is steep (>∼1) over a sufficient range of diastolic intervals, a stable beat-to-beat APD oscillation can occur, called electrical alternans. If the amplitude of these APD alternans grows large enough, AP failure occurs, causing conduction block and arrhythmia induction (140, 141). APD alternans have been mechanistically linked to the genesis of T-wave alternans that appear on the ECG (142) and have been shown to be a good marker of risk for sudden cardiac death in patients (143). APD alternans may be spatially concordant, when all regions of tissue alternate in phase, or spatially discordant, when adjacent regions alternate out of phase, separated by a nodal line at which no alternans occurs (144, 145). Discordant alternans is more arrhythmogenic than concordant alternans because it can amplify the spatial dispersion of repolarization enough to cause unidirectional conduction block and the onset of reentry (142, 146–148).

In addition to APD restitution, beat-to-beat alternation in the amplitude of the CaT can also independently lead to APD and repolarization alternans (149, 150). According to this mechanism, repolarization alternans arise when the HR exceeds the capacity of myocytes to cycle intracellular Ca^2+^. At fast rates, both refractoriness of SR Ca^2+^ release (due to refractoriness of RyR) and SERCA Ca^2+^ reuptake can lead to beat-to-beat alternation in the amplitude of the CaT. CaT alternans, in turn, can result in cellular APD alternans through several electrogenic Ca^2+^-sensitive sarcolemmal currents. Reduced SERCA expression (151) and/or function (152) have been linked to the genesis of CaT alternans, as well as interventions that modify RyR Ca^2+^ release (153–156).

Regardless of the underlying mechanism governing alternans in a particular experimental model, the slower the pacing rate at which alternans emerge and/or the larger the magnitude of APD or CaT alternans, the more susceptible a heart may be to reentrant arrhythmias. Likewise, the presence of spatially discordant alternans is typically more arrhythmogenic due to large gradients of repolarization (142, 146–148). In addition to the pacing threshold, alternans magnitude can be quantified directly by comparing the absolute difference in APD between long and short beats (and/or relative amplitude of large and small CaTs). However, APD may only vary by a few milliseconds, which may be difficult to accurately detect. Therefore, algorithms in the frequency domain [similar to those used clinically to detect micro-volt T-wave alternans (157)] have also been adapted for use with experimental data (155, 158).

##### Wavelength for reentry.

Another important parameter that determines whether a reentrant circuit can occur and/or the number of reentrant waves that can be present in the myocardium is called the wavelength for reentry (159). Wavelength is the product of the effective refractory period and CV. Wavelength determines the minimal physical size of reentry and, for reentry to be self-sustained, wavelength must be less than the physical size of the myocardium. The shorter the wavelength, the more likely it is that one or more reentrant circuits can be maintained. Notably, myocardial tissue size can impact the initiation and stabilization of arrhythmias (55, 58, 160–162). Accordingly, reentry and fibrillatory activity are less commonly observed in smaller rodent models, unless other factors are present to increase tissue heterogeneity (e.g., ischemia, fibrosis, ion channel over-/underexpression) (163, 164).

##### Arrhythmia complexity and organization.

Although monomorphic and polymorphic tachycardias can be associated with reentry, automaticity, or triggered rhythmic activity, either alone or in various combinations, fibrillation is typically driven by multiple reentrant circuits that spawn wandering wavelets. In monomorphic tachycardia, the shape of each beat on the ECG looks the same because the impulse is either being generated from a single, stable point, or due to a stable reentrant circuit. Polymorphic tachycardia can be caused by the focal activity of varying origins and propagation patterns (repetitive firing of multiple sites) or nonstationary (drifting), vortex-like (spiral) reentrant activity. Analysis of the dominant frequency of arrhythmic activity can be useful to distinguish between these mechanisms and to characterize the complexity of arrhythmic activity (165, 166). For electrode array or optical mapping data, at each location, a Fourier transform of the signal is computed and the dominant frequency for that location is identified and mapped (167). A spatially homogeneous dominant frequency map suggests a more organized, monomorphic tachycardia, whereas more complex and higher-frequency maps indicate fibrillatory activity.

The spatiotemporal organization of complex electrical activity can also be analyzed using a phase plane transformation of mapping data (168, 169). This approach is particularly well suited for analyzing electrical rotors and the breakup and collision of electrical wavefronts (170). In one implementation of this method (delay embedding), signals at each recording site [e.g., *V*_m_(*t*) at each pixel in an optical mapping data set] are plotted against a time-shifted version of the same signal [e.g., *V*_m_(*t* + Δ*t*), where Δ*t* is a time delay] to generate a phase portrait (171). The angular position of each time point in the phase portrait is the phase in the activation-recovery cycle of the mapped location at that time (171–173). In cardiac phase maps, phase singularities denote a rotating wavefront, or a wave break, and are identified as being surrounded by sites that exhibit a continuous 360° phase progression. The number and activity (e.g., stationary or drifting) of phase singularities quantify the spatiotemporal organization of complex arrhythmias. In addition to delay embedding, there are other ways to compute phase, therefore the specific method should be reported to ensure reproducibility.

##### Focal activity.

In contrast to reentrant arrhythmias, focal tachycardias are associated with one (monomorphic) or several (polymorphic) local sources of automaticity. Focal activity can be either ectopic automaticity (enhanced normal automaticity from latent pacemakers or abnormal automaticity from working myocardium) or triggered activity [associated with either early or delayed afterdepolarizations, EADs, or DADs (174)]. DADs occur during diastole, whereas EADs occur before repolarization is complete. EADs typically occur when APD is prolonged because of decreased K^+^ currents and augmentation of either the Ca^2+^ or Na^+^ current. EADs can appear without the involvement of intracellular Ca^2+^ activity; however, elevated Ca^2+^ can facilitate the development of EADs. Bradycardia, long pauses, and APD prolongation, therefore, provoke EAD initiation. In contrast, DAD-induced triggered activity commonly underlies arrhythmias precipitated by tachycardia. Mechanistically, DADs are associated with the spontaneous release of Ca^2+^ from the SR secondary to cellular Ca^2+^ overload. This spontaneous SR Ca^2+^ release activates a transient inward current (*I*_ti_, via forward mode *I*_NCX_), which can trigger an AP (131).

Focal arrhythmias can be characterized via the localization of ectopic foci, their frequency, and spatial-temporal characteristics of myocardial activation. Source-sink interactions are critical determinants of whether cellular-triggered activity can propagate to the surrounding myocardium. Smaller cardiac tissue sizes and ∼one- and ∼two-dimensional structures (e.g., ∼1-dimensional Purkinje fibers or trabeculae, or ∼2-dimensional layers such as thin walls of rodent hearts) are much more susceptible to ectopic activity compared with three-dimensional volumes due to the greater current sink in the larger tissue volumes (175). Notably, pathological conditions associated with heart failure, ischemia, or gap junction uncoupling can significantly reduce the current sink, wherein a modestly sized cell population can be sufficient to generate ectopic beats (132, 175–177).

## EXPERIMENTAL APPROACHES

A variety of experimental approaches can be applied to assess the aforementioned parameters.

In this section, we discuss the techniques commonly used for in vivo and ex vivo electrophysiological studies and their suitability for the assessment of different electrophysiological parameters. Special considerations and unique challenges in each technique, as well as advantages and limitations, are also discussed (see Table 1).

**Table 1.** Electrophysiological measurement techniques along with common outputs and strengths and limitations of each method

Technique	Common Outputs	Strengths	Limitations and Pitfalls
ECG telemetry	• R-R interval • HRV parameters • Arrhythmias: spontaneous, drug-induced	• Freely moving/behaving animals • Long-term recordings over days/weeks/months • Day-night rhythms	• Surgery required for telemeter implant • Expensive hardware • Low SNR prohibits detailed ECG analysis • Large amount of data
ECG in conscious unrestrained animals	• R-R interval • ECG parameters (PR, QRS, QT) if adequate SNR • Arrhythmias: spontaneous, drug-induced	• Longitudinal studies (serial noninvasive recordings)	• Animals must be trained to stay on footpads • Low SNR may prohibit detailed ECG analysis
ECG in conscious restrained animals	• R-R interval • ECG parameters (PR, QRS, QT) if adequate SNR • Arrhythmias: spontaneous, drug-induced	• Suitable for acute drug injections • Longitudinal studies (serial noninvasive recordings)	• Need to train/acclimate animals
ECG in conscious tethered animals	• R-R interval • ECG parameters (PR, QRS, QT) if adequate SNR • Arrhythmias: spontaneous, drug-induced	• Freely moving/behaving animals • Longitudinal studies (serial recordings)	• Surgery required for electrode implant • Tethered wires
ECG in anesthetized animals	• ECG parameters (RR, PR, QRS, QT) • Arrhythmias: spontaneous, drug-induced • With programmed stimulation: pacing-induced arrhythmias, ERP (ventricular, atrial, AVN), SNRT	• Suitable for acute drug injections • Longitudinal studies (serial recordings) • Programmed stimulation can be performed (transesophageal or intracardiac) • Can be combined with intracardiac electrograms	• Anesthetic effects can impact electrophysiology
Intracardiac electrogram in anesthetized animals	• Arrhythmias: spontaneous, drug-induced • With programmed stimulation: pacing-induced arrhythmias, ERP (ventricular, atrial, AVN), SNRT	• Suitable for acute drug injections • Programmed stimulation can be performed	• Anesthetic effects can impact electrophysiology • Terminal procedure
Ex vivo ECG in isolated hearts	• ECG parameters (RR, PR, QRS, QT) • Arrhythmias: spontaneous-drug-induced • With programmed stimulation: pacing-induced arrhythmias, ERP (ventricular, atrial, AVN), SNRT	• Can be combined with microelectrode, MAP, or optical recordings • Suitable for studying drug effects	• ECG morphology can depend on electrode placement • Loss of hormonal and autonomic regulation
Microelectrode recordings	• AP parameters (APD, AP amplitude, upstroke velocity, restitution) • Absolute *V*_m_	• Precise, absolute measurement of cardiomyocyte *V*_m_	• Limited number of recording sites—little spatial information • Contractile motion may produce artifacts (excitation-contraction uncouplers may or may not be needed)
Monophasic action potential (MAP) recordings	• AP parameters (ADP, relative AP amplitude, relative upstroke velocity, restitution) • Relative *V*_m_	• Readily applied to beating heart (may be used in vivo)	• Limited number of recording sites—little spatial information • Relative *V*_m_
Multi-electrode arrays	• Activation time, repolarization time, activation-recovery interval • 2-D maps of activation/repolarization, CV • Arrhythmia susceptibility & spatial characteristics	• Readily applied to beating heart • Abundant spatial information	• No AP properties, only activation & repolarization • Large amount of data
Optical mapping	• AP parameters (APD, relative upstroke velocity, restitution) • 2-D maps of activation/repolarization, CV • Arrhythmia susceptibility & spatial characteristics • Ca^2+^ transient properties (if measured)	• Abundant spatial information • Can be combined with ECG, electrode recordings, & Ca^2+^ imaging • Immune to electrical artifact (defibrillation studies possible)	• Contractile motion produces significant artifacts—excitation-contraction uncouplers or intensive postprocessing required to eliminate motion artifact • Large amount of data

AP, action potential; APD, action potential duration; AVN, atrioventricular node; CV, conduction velocity; ECG, electrocardiogram; ERP, effective refractory periods; HRV, heart rate variability; SNR, signal-to-noise ratio; SNRT, sinus node recovery time; *V*_m_, transmembrane voltage.

### In Vivo ECG Telemetry

Implantable telemetry allows researchers to assess HR, quantify standard ECG parameters, detect arrhythmias, and monitor other variables including body temperature, activity, arterial blood pressure, the electroencephalogram, the electromyogram, and respiration in a wide range of animal models, including rats and mice (156, 178–185). A major advantage of telemetry is that physiological variables are measured in freely moving animals that behave normally in their cages, an important distinction from other in vivo measurements described later (*Acute In Vivo ECG Recording*). Another advantage is that data can be continuously collected over days, weeks, or months. Long-term ECG recordings allow for investigations of time as a biological variable, including studies of ECG changes during the light or dark cycle (186), biological time (i.e., the time relative to the start of the light) (187), or circadian time (time in constant conditions) (188–190). Telemetry can also be used to determine the impact that changes in the environment or behavior have on physiological variables (191–195), including temperature, activity, feeding, diet, and exposure to environmental compounds (185, 196).

Telemetry is important for assessing an arrhythmia phenotype, particularly for arrhythmias that occur spontaneously or develop over time, as may occur with atrial arrhythmias that first manifest as atrial extrasystoles and then progress to paroxysmal atrial fibrillation (197). Telemetry may also be ideally suited for the observation of arrhythmias triggered during physical activity and allows HRs during stress to be compared with undisturbed resting HR (198). Arrhythmias triggered during sleep and bradycardia are ideally observed with telemetry if the animals are allowed to rest undisturbed (199).

Telemetry is also used to measure HRV (85, 200), as mentioned earlier in *Characterization of the Sinus Node*. HRV is an assessment of autonomic balance and is higher during enhanced parasympathetic tone and lower during enhanced sympathetic tone (201). HRV is quantified using time-domain measurements to calculate RR interval variability within a given time interval. Spectral analyses of RR intervals also measure HRV using spectral power measured in high- and low-frequency bands, where the bands are determined by the animal model (202). Additional details for HRV metrics are provided in prior reviews (83, 203, 204). Heart rate recovery (HRR) is another assessment of autonomic tone that measures parasympathetic activity after physical exertion (205, 206). HRR is measured in rodents using ECG telemetry and a treadmill running protocol. After reaching a designated level of treadmill running effort, the treadmill is stopped and the drop in HR is monitored for a specified interval. A smaller HR drop (lower HRR) has been associated with reduced vagal capacity and tachycardia in mice (207) whereas greater HRR was consistent with improved cardiac function in treated heart failure rats (208).

**Figure 7.**
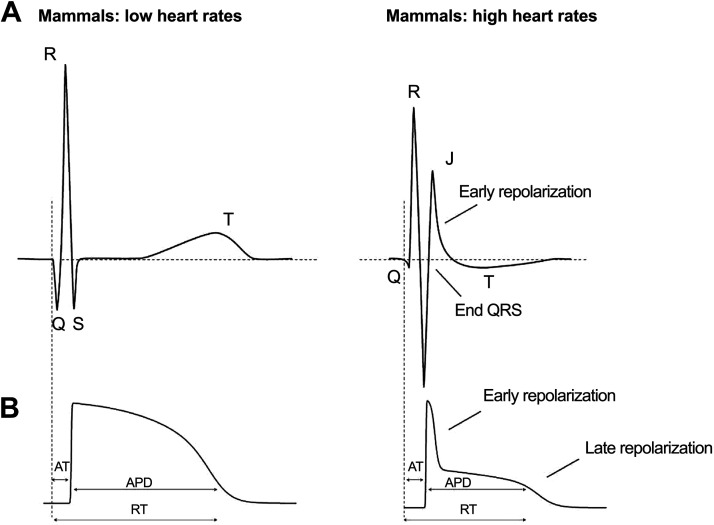
Relationship between electrocardiogram (ECG) and action potential (AP) morphology in mammals with low heart rates (*A*) vs. mammals with high heart rates (i.e., rodents) (*B*). Reproduced with permission from Ref. 213.

Reproducibility is a concern for telemetry studies, often driven by incomplete descriptions of study design, methods, and analysis used to calculate the derived measures (209, 210). Reproducibility is also impacted by the experimental challenges of using telemetry (211). A major issue remains the lack of a consensus range for ECG parameters in both anesthetized and conscious free-moving animals. This is particularly true for mouse studies. There is a large amount of interobserver variability in the quantification of QRS and QT intervals in mice, and a change in the QT interval duration as a function of mouse heart rate is seen in some studies, but not others (101, 187). Such interobserver differences in ECG measurements in rodents like mice and rats are likely caused by the relatively large amplitude and overlapping J waves and low amplitude T waves (212) (Fig. 7). More specifically, in mice, the S wave is immediately followed by a J wave that represents repolarization and should not be included in the calculation of the QRS duration. Instead, the end of the QRS complex should be calculated by the point where the S wave intersects with the isoelectric line (214). Additional factors that contribute to interobserver differences in ECG parameters between studies include the strategy used to quantify intervals (manual vs. automated) and strain- or sex-specific differences.

Other challenges for telemetry studies include the requirement for expensive hardware that often has limited battery life. Surgery is required to implant the devices and animals need several days to recover (215). In rodents, HR and HRV measurements can take ∼10 days to stabilize after major surgery (204). Acute drug injections can require the handling of conscious animals, which may cause animal stress. Care should be taken when comparing measurements between studies, as ECG and HRV indices can vary significantly based on the length of a recording and the time of day the ECG is acquired.

Telemetry experiments can quickly produce a large amount of data, and as such, data management and analyses are not trivial. Challenges to analyzing the acquired signals include low signal-to-noise ratio, animal motion artifacts, and changes in the orientation of the ECG leads during an experiment. Motion artifacts and the lack of a fixed lead position prohibit ECG wave amplitude comparisons between or within subjects. Movement of the ECG leads can also cause inverted ECG signals, variation in the signal-to-noise ratio, and intermittent loss of signal, making the beat-to-beat measurements or detection of spontaneous arrhythmias challenging. Distinguishing artifact from arrhythmia is not always easy and it is not uncommon for some investigators to misidentify artifact as a possible arrhythmia. Some of the challenges of telemetry experiments are mitigated by proper study design. For example, the battery life of an implanted device can be conserved by limiting the number of variables recorded simultaneously or with intermittent recordings (the devices are easily turned on and off). Signal averaging, if appropriate, will increase the signal-to-noise ratio. Removal of nearby devices that might cause electrical interference and Faraday shielding between cages can also improve ECG signal quality. Commercial or custom-developed software programs can be used to analyze a large amount of data, as well as limit the impact of signal artifacts.

### Acute In Vivo ECG Recording

Although telemetry remains the gold-standard approach for conscious ECG recording in animals, there are a number of limitations (as mentioned in *In Vivo ECG Telemetry*) that motivate use of alternative methods.

#### In vivo ECG recording in conscious, unrestrained animals.

Nontelemetric approaches have been developed for ECG recordings in conscious, unrestrained animals using a footpad-based system in which animals are placed on a platform containing footpad electrodes that facilitate the acquisition of ECG signals via the paws (211, 216, 217). These systems can be used with a number of animal models (e.g., mice, rats, guinea pigs) as long as the platform is large enough and the footpad electrodes can be properly spaced to accommodate animals of different sizes. Footpad-based systems can be used to measure HR, standard ECG intervals, and for arrhythmia detection. These systems are relatively easy to set up and use and are ideal for longitudinal studies (i.e., developmental, aging, disease progression, or chronic drug studies) as measurements can be obtained in the same animals noninvasively and serially over time.

There are, however, some disadvantages to this approach. Animals must be trained to remain on the platforms, which are generally designed to accommodate a single animal. Although conscious and unrestrained, the animal must remain in position on the footpads to acquire signals. Thus, the animals are unable to walk freely, groom, etc. Furthermore, movement off the footpads will result in disruptions in ECG acquisition. Although acute drug injections are possible, this requires handling of the animals, which may be a confounding factor. Footpad-based systems are also more susceptible to noisy recordings, although noise-reducing technologies are available. These systems require continuous monitoring by the investigator, which typically means studies are conducted during the daytime when nocturnal animals, such as mice, are less active. Such daytime recordings can have implications for studies involving circadian aspects. Because of these limitations, these systems are best suited for studies requiring periodic, short-term (i.e., a few seconds to minutes) ECG screening.

#### In vivo ECG recording in conscious, restrained animals.

ECGs may also be recorded in conscious animals placed in restraining devices such as plastic tubes or custom-made elastic jackets that fit the thoracic circumference of the animal and hold them in a fixed position (218–220). These devices are designed to allow attachment of ECG electrodes to the limbs, to reduce motion using a footpad-based recording system, and/or to provide access for acute drug injections via the intraperitoneal cavity or the tail vein (28, 221). Restraining systems can be designed to accommodate animals of different sizes. One of the disadvantages of restraining systems is the need for training to acclimate the animals to the device. Even with training, being restrained and unable to move may induce stress responses. Additional handing may be required for acute drug studies, which may further complicate this issue. It is essential that the restraining device does not interfere with respiration. As with the footpad-based system discussed earlier, studies require continuous monitoring. Because of these limitations, these systems are also best suited for studies requiring short-term ECG screening.

#### In vivo ECG recording in conscious, tethered animals.

A tethered ECG system implants wired electrodes that are tunneled under the skin via a midscapular incision and positioned near the four limbs of the animal (211, 222). The advantage of this system is that it enables the animals to move freely within their cages, ensuring access to food and water (as long as the ECG wires are sufficiently long). Because the electrodes are implanted, the ECG signals are not affected by animal movement enabling longer recordings without interruption in signal acquisition. A disadvantage of this system is that the setup requires initial surgery to implant the electrodes. Also, although the wires can be set up to remain out of reach of the animal, it is best that the animals are not left alone during recording, which creates similar limitations to the two systems described earlier. Tethered ECG recordings are best used for periodic recordings that are intermediate (i.e., minutes) in duration.

### In Vivo Recording and Arrhythmia Provocation in Anesthetized Animals

ECG recording in conscious animals is often preferred, as anesthetic agents can depress heart rate, blood pressure, autonomic regulation, and respiration (211, 217, 220, 223). However, the variable signal-to-noise ratio associated with conscious recordings can limit analysis to heart rate or HRV measurements, as these parameters are only dependent on the detection of prominent R waves. ECG recordings with superior signal quality can be recorded in anesthetized animals using either a footpad or subcutaneous needle electrodes (90, 216). For the latter, electrodes are typically positioned for lead I (right and left forelimb) and/or lead II (right forelimb, left hindlimb). More invasive catheter-based electrogram recordings with or without programmed electrical stimulation can also be performed in anesthetized animals.

With all these approaches, the choice of anesthesia needs to be carefully considered. Inhalable isoflurane and injectable ketamine-xylazine cocktail are the most commonly used anesthetic agents for in vivo ECG and programmed electrical stimulation studies. Isoflurane is typically preferred because of its minimal impact on cardiac function and the relative ease of controlling anesthesia depth and duration (224). However, isoflurane administration requires a specialized vaporizer and a proper scavenge system, which needs regular calibration and inspection. On the other hand, ketamine-xylazine can induce bradycardia, may be insufficient to produce a surgical plane without an analgesic agent (if needed for invasive approaches), and additional doses are often needed to maintain the surgical plane for extended study duration (225, 226). Proper control of body temperature is another critical consideration during anesthetized recordings, as both hyperthermia and hypothermia can influence the inducibility of arrhythmias and cardiac conduction properties. Therefore, a heating pad with a temperature monitor is required. An integrated heating and surface ECG recording board are also recommended.

**Figure 8.**
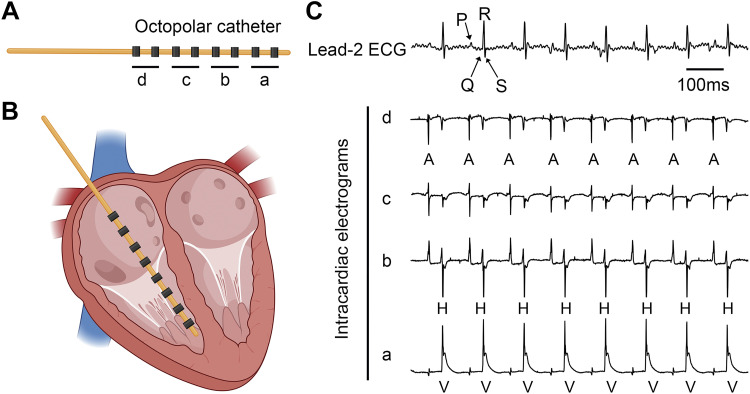
Intracardiac catheterization for programmed electrical stimulation. *A*: designation of four bipolar leads on an octopolar catheter. *B*: placement of the catheter inside the heart. *C*: representative simultaneous recording of surface electrocardiogram (ECG) (lead 2) and intracardiac electrograms from four bipolar leads (A, atria, H, His bundle, and V, ventricle). Created partially using Biorender.com and published with permission.

As spontaneous arrhythmias are often difficult to observe in small animals, pharmacological “stress tests” may be performed using the β-adrenergic agonists isoproterenol or dobutamine, or more specific pharmacological arrhythmia provocation can be performed. For example, isoproterenol is often combined with caffeine if SR Ca^2+^ handling abnormalities are suspected because caffeine will sensitize RyR to spontaneous Ca^2+^ release (227, 228). Another method to provoke arrhythmias (or assess electrophysiological parameters such as SNRT, Wenckebach periodicity, AVNERP, VERP, etc.; see quantifying electrophysiology for details) is with the use of in vivo pacing (or programmed electrical stimulation) (90, 229, 230). Programmed electrical stimulation can be performed using either transesophageal pacing or intracardiac pacing, which relies on the intravenous delivery of a pacing catheter in anesthetized animals (129, 231). For intracardiac pacing, a catheter is placed inside the right side of the cardiac chambers via the external jugular vein (90, 129, 232). Based on the waveforms of intracardiac electrograms sensed by the electrodes at the tip of the catheter, the placement of the catheter can be determined and adjusted if necessary (Fig. 8). The advantage of intracardiac pacing is the ability to simultaneously record the surface ECG and multiple intracardiac electrograms from the right atrium, AVN, His bundle, and right ventricle (129, 229). The intracardiac electrograms can aid in the determination of AV conduction and in assessing susceptibility to pacing-induced arrhythmias.

Typically, transesophageal pacing is performed using a 2-Fr octapolar catheter. Because the induction of certain types of arrhythmias, such as atrial fibrillation (AF), can vary in control and AF-susceptible animals depending on age, sex, strain, and pacing protocol used (23), it is crucial to optimize these parameters initially for each model under study to enhance reproducibility. A complication of transesophageal pacing is inadvertent parasympathetic stimulation, manifested by exaggerated pacing-mediated AV block and AF induction, due to stimulation of ganglionic plexi on the posterior left atrium. For additional insight into this effect, measurements can be made with and without full blockade of the autonomic nervous system (233). This complication occurs most likely during pacing at high stimulus intensities and short cycle lengths (23). Therefore, it is essential to achieve the lowest possible capture stimulus amplitude during threshold determination (with careful catheter positioning, a threshold of ≤0.75 mA at 2 ms is usually sufficient), with pacing conducted at twice the threshold. Animals experiencing excessive AV block [i.e., bradycardia for ≥10% of a single pacing train duration (23)] associated with artifactual AF induction should be excluded from the analysis. One advantage of transesophageal pacing over intracardiac pacing is that it is a less invasive and survival procedure, which allows for serial testing in a given animal.

To assess susceptibility to atrial arrhythmias, cumulative pacing-mediated AF duration (or burden) or a binary AF occurrence per animal (e.g., defined as AF during 2 of 3 pacing trains) can be quantified. Induction of AF episodes [most commonly ≥1 s (234–240)] should be assessed in both sexes and over a range of ages. In some studies, male mice displayed AF susceptibility while females did not (23). In addition, genetic models and control strains typically develop AF inducibility at different rates with increasing age (241, 242). Therefore, studies should be performed during a window when the phenotype is inducible in a given disease model, but not routinely inducible in controls. Interestingly, for a specific animal model, AF susceptibility may be increased with one mode of pacing (i.e., burst S1-S1 or decremental S1-S2) but not the other, while induction can vary with the number of pacing trains and shortest pacing cycle length used. Thus, pilot studies should be performed to optimize the pacing protocol for each AF model. To enhance reproducibility, studies should be performed within a several-hour time period each day, with a recovery interval of at least 30 s between pacing trains (234, 237, 238).

Programmed electrical stimulation can also be performed to induce ventricular tachyarrhythmias, such as ventricular tachycardia (VT) and ventricular fibrillation (VF). Similarly, the incidence and duration of VT or VF can be quantified upon different pacing protocols (e.g., burst pacing or extrastimuli). In certain genetic models, such as the catecholaminergic polymorphic ventricular tachycardia mutant mice, injections of epinephrine and caffeine may be needed to mimic β-adrenergic stimulation before pacing (243–245).

### Ex Vivo Electrode-Based Techniques

Although in vivo electrophysiological recordings provide assessments in the most physiologically relevant conditions, highly detailed measurements of APs, conduction properties, and arrhythmia mechanisms often require excised perfused hearts and tissues. The Langendorff-perfused heart (246) is the most used ex vivo preparation for tissue-level experiments. Isolated coronary perfused ventricular wedge preparations (247, 248), superfusion of small cardiac tissue preparations [e.g., excised atria, trabeculae, or papillary muscles (249)], and even modified Langendorff perfusion with intact innervation are also extremely useful (250–252). For preparations that are only superfused, the tissue must be sufficiently thin (<∼0.5 mm) for the diffusion of oxygen and nutrients, as thicker tissue will quickly become ischemic (253, 254). The atria of rodents and rabbits can typically be maintained with only superfusion (255), whereas the thicker ventricles of rabbits and larger species require coronary perfusion (247).

Important variables to be considered for ex vivo studies include the type of anesthesia and euthanasia used before tissue excision, the maintenance of adequate coronary flow and pressure during perfusion, and the temperature, composition, pH, and oxygenation of the perfusate (256–258), as recently reviewed (259). Tissue contraction may introduce motion artifact in some recording techniques, such as microelectrode recordings and optical mapping. To overcome this, an excitation-contraction uncoupler is typically administered to the tissue to prevent contraction (260), but the cessation of contraction removes the effect of mechanical stretch (261) and dramatically reduces metabolic rate (262, 263), both of which may alter the electrophysiological state of the tissue. Therefore, experimental design and data interpretation must take these factors into account. We have recently suggested best-use practices for one of the most commonly used excitation-contraction uncouplers, blebbistatin (262).

#### ECG in isolated hearts and tissues.

Although more commonly thought of as an approach for in vivo measurement of whole heart electrical activity, the ECG is also often acquired from the isolated heart and tissue preparations. At least two electrodes, an anode, and a cathode are placed at two distinct positions on or near the preparation. A third electrode could be placed in the bath to serve as a common ground to minimize common mode content, such as pacing artifacts. The relative potential difference between the anode and cathode is then acquired. From this, a representation of the overall electrical activity between the electrodes is generated, which is useful for monitoring the electrical features and rhythm of the tissue. This approach is useful in conjunction with localized electrophysiological measurements [e.g., microelectrodes or monophasic AP (MAP) recordings, described later], to determine their relation to global electrical activity (264) and to monitor acute responses to physiological perturbations (265), including monitoring tissue viability after administering an excitation-contraction uncoupling agent (260).

Although many of the considerations for acquiring and interpreting ex vivo and in vivo ECG signals are the same, there are important differences. One is that the electrodes are different. For ex vivo measurements the electrodes can be *1*) spring-loaded pellet electrodes that are pressed against the tissue (266, 267); *2*) needle or hook electrodes that are inserted into the tissue; or *3*) when the preparation is in a bath filled with an ionic solution, disk or needle electrodes that are positioned close to, but not in contact with, the tissue will acquire the electrical activity conducted through the solution (103, 268). In all cases, the ECG electrodes must be positioned some distance from each other so that a potential difference exists and can be detected. Another difference is that ex vivo signals will have much lower amplitude and will require amplification. Finally, the morphology of ex vivo signals will be highly dependent on electrode position. Although some positions may produce ECG signals that resemble common in vivo configurations, many will not (214), so their interpretation (i.e., periods of activation and repolarization) must be carefully considered and may be greatly affected if shifts in electrode position relative to the tissue occur during an experiment.

#### Microelectrode recordings.

Soon after the invention of the ECG, electrophysiologists began developing instrumentation to measure electrical activity from single cardiomyocytes embedded in cardiac tissue (269). Sharp-glass micropipette electrodes or “microelectrodes” were first used in 1904 to capture a single bacterium in live cells (270) and later applied by Ida Hyde (the first female member of the American Physiological Society) for electrical stimulation of living cells (271). Microelectrodes were then used by Graham and Gerard (272) to record the resting *V*_m_ of frog muscle fibers. The technique was later improved by Ling and Gerard (273), and the use of “Ling-Gerard” electrodes has been the gold standard for the measurement of single-cell transmembrane potential from intact tissue ever since (269).

Sharp microelectrodes used for the measurement of cardiomyocyte *V*_m_ are typically pulled from borosilicate glass with tip diameters of 0.5 μm or less, are filled with 3 M KCl solution, and are held by specifically designed holders with an internal electrode wire connected to a headstage and amplifier. They should be advanced into the tissue by precise three-axis micromanipulators, and when the tip penetrates a cell membrane, the lipids in the membrane adhere to the glass, creating a high-resistance seal that allows for the measurement of the potential difference between the interior and exterior of the cell. As such, microelectrodes provide the most precise measurement of *V*_m_ from single cells within a multicellular cardiac preparation (274). In contrast to surface electrograms or optical mapping, microelectrodes allow for the measurement of absolute *V*_m_, so that the resting membrane potential, upstroke velocity, and amplitude of the AP can be quantified. Absolute *V*_m_ is critical for comparing measurements across groups where nonrelative *V*_m_ values are important, for instance in situations where changes in resting *V*_m_ are expected [e.g., ischemia (275), decremental conduction (276), or altered electrical coupling (277, 278)]. Moreover, microelectrode-based APs can be measured without excitation-contraction uncoupling and has been applied to contracting preparations (264, 279).

Although sharp-glass micropipette electrodes allow for precise, absolute measurement of cardiomyocyte *V*_m_ in intact tissue, their use is limited by their localized nature. Recordings cannot practically be made simultaneously across many sites, to simultaneously record APs throughout the tissue. Since a limited number of microelectrodes can be used at a time, this approach prevents the study of AP propagation and complex spatial-temporal processes, such as ectopic excitations, leading pacemaker shift, or reentrant arrhythmias. The use of microelectrodes is also generally limited to excised myocardial tissue preparations, monolayers, or single cells, as a large amount of movement with contraction often does not allow for their use in the whole heart [without the use of excitation-contraction uncouplers (261)], although some have had success overcoming this limitation using detachable “floating” microelectrodes in active moving organs (264). Of note, microelectrode recordings can also suffer from substantial electrical artifacts, for instance during or immediately after electric shocks used for defibrillation, so are not appropriate for certain experimental settings.

#### Monophasic action potential recordings.

The recording of AP in contracting whole heart preparations is possible with extracellular potential measurements from the surface of the heart, which allow for the acquisition of the monophasic AP (MAP). The first MAP was recorded in 1882 by Burdon-Sanderson and Page, who placed one electrode on the intact epicardial surface and the other on an injured site of the frog heart and thereby captured the phasic electrical changes of the cardiac cycle (280). In 1934, suction electrodes were introduced for MAP recording (281), and later it was determined that the MAP can be obtained by simply pressing one electrode against the epicardium with another electrode touching the surface nearby (282). The MAP is now typically measured with an extracellular electrode (∼0.25–1.5 mm in diameter) (283–285) and generates a signal that represents the flow of current between inexcitable cells directly under the electrode (where contact pressure has depolarized them to between −30 and −20 mV, making them refractory) and adjacent excitable cells (286). During the AP, the excitable cells depolarize as normal, while the refractory cells maintain their potential, with the recorded MAP reflecting the potential of the depolarized cells coupled to the volume of inexcitable cells.

In this way, the MAP reproduces the time course of an AP from a localized area on the surface of the heart with high fidelity, similar to that recorded by microelectrodes (284). MAP recording has a distinct advantage over microelectrode recordings, however, as they can be readily applied to the beating heart, including in in vivo open-chest animals (287, 288). Unlike microelectrode recordings, though, the MAP does not provide absolute values of *V*_m_. Instead, they are useful for assessing relative changes in the AP, as long as a stable baseline recording is obtained before and after an intervention (286). MAP recordings are also useful for detecting proarrhythmic changes in the AP (284, 289), but stringent quality criteria to the MAP recording must be applied when interpreting electrical abnormalities, as signals that appear to be EADs or DADs can also result from unstable electrode contact.

Even in the relatively small mouse heart, MAPs can be recorded from the atria (290) and ventricles (284, 290–292), and have been used to assess electrical restitution (126), but electrode size should be carefully considered. Since a MAP probe measures the electrical current from cells at the surface and depolarized cells underneath, larger MAP probes that measure this over a larger volume of cells will blur the AP upstroke and initial rapid (*phase 1*) repolarization, and artificially prolong the later (*phase 3*) repolarization. Elastic probes with an electrode tip diameter of 0.25 mm are recommended for mouse hearts but should be used carefully as small probes may puncture the myocardium (284).

#### Multielectrode arrays and microelectrode arrays.

Although sharp glass micropipette electrode and MAP recordings provide an accurate representation of the AP from a single cell or a small group of cells, their application is limited to localized measurements and cannot be used to map the sequence of depolarization and repolarization from tissue. To overcome this, arrays of extracellular unipolar electrodes are placed in contact with the tissue. The extracellular potential is acquired simultaneously from each electrode using a multichannel data acquisition system. Tissue depolarization and repolarization times are extracted using the extracellular potentials of each electrode to map conduction, repolarization, and to estimate APD by computing the interval between activation and recovery (also known as the activation-recovery interval) (293). Large arrays of 504 electrodes have been used to map ventricular arrhythmias in large animals (294) whereas smaller arrays of up to 64 electrodes have been used to map mouse hearts (295–297) and the His bundle of rabbit hearts (298).

The advantage of cardiac mapping using multielectrode arrays is that it can be used to study contracting hearts in vivo or ex vivo, as blood does not interfere with signal acquisition, and the arrays can be placed on structures (e.g., His bundle) that cannot be accessed by optical mapping (298). Mapping data are analyzed to study excitation wavefront propagation during sinus rhythm, electrical pacing, and during complex arrhythmogenic events, including premature beats and focal and reentrant arrhythmias. In addition to measuring CV, the spatiotemporal dynamics of electrical activity can be analyzed using the cardiac phase (168) (see, *Arrhythmogenesis*) and the lifetimes of individual wavefronts and their fractionations and contact with other wavefronts can be tracked using directed graph (299, 300). Extracellular electrode array innovations have enabled the in vivo estimation of cardiomyocyte transmembrane current (301) as well as tissue macro and microimpendences (302, 303), which are important electrical properties of the cardiac syncytium. Despite the many advantages of extracellular recording with multielectrode arrays, care must be taken when interpreting local repolarization in signals from rodents. Due to the triangular shape of the AP, there may not be a clear isoelectric segment between activation and repolarization deflections (214, 304).

### Ex Vivo Optical Mapping in Isolated Hearts and Tissues

Optical mapping relies on fluorescent indicators that transduce physiological phenomena, including *V*_m_ and intracellular Ca^2+^, into an optical signal that is often recorded using a high-speed camera. Fluorescent indicators may be exogenously delivered to cells, where they localize to cell membranes or specific subcellular compartments (e.g., mitochondria or SR). Cells can also be genetically encoded to produce voltage or Ca^2+^-sensitive indicators, which avoid some limitations of exogeneous indicators (e.g., phototoxicity), are useful for long-term studies, and enable measurements from specific cell types (305–308). Excitation-contraction waves can also be optically mapped using dye-free approaches (309, 310) and in combination with fluorescent indicators and ultrasound imaging (311). Here, we will focus on mapping approaches using exogenously delivered *V*_m_ and/or Ca^2+^ indicators because of their widespread use in a variety of animal models (312, 313).

All fluorescent indicators absorb photons at one wavelength (excitation spectrum) and emit photons at a longer wavelength (emission spectrum). The optical signal is encoded by changes in the emission spectrum that are caused by a specific physiological phenomenon. For Ca^2+^-sensitive indicators, the emission spectrum amplitude increases in response to the indicator binding with Ca^2+^, resulting in increased fluorescence. For many *V*_m_-sensitive indicators, the peak wavelength of the emission spectrum shifts slightly (the light emission changes color) in response to membrane depolarization, resulting in a change in fluorescence. The fluorescent indicator, optical filters, lens objectives, and mirrors should be selected to maximize both the excitation light that energizes the indicator and the emitted light that carries the signal, while also minimizing the overlap of excitation and emitted light. For example, a broad-spectrum excitation light source may maximally excite an indicator, but excitation light may reflect back through the emission filters to overwhelm the optical signal(s) of interest.

When simultaneously imaging more than one physiological phenomenon, indicators that have similar excitation spectra are often used together because the same excitation light will energize each indicator, eliminating the need for another light source, as when imaging APs with RH237 and CaTs with Rhod2-AM (314, 315). To reduce optical cross talk between simultaneously imaged indicators, it is imperative to choose indicators that have emission spectra that do not overlap or only minimally overlap. Published reviews on fluorescent indicators, with recommended filter sets, provide a wealth of information for the optimal design of an experiment to optically map more than one indicator (130, 308, 315, 316). In unique situations where optical cross talk is unavoidable, it is theoretically possible to computationally correct for signal overlap. However, this assumes that the emitted light and associated optics produce a linear relationship between the acquired optical signal and the transduced physiological phenomena, which may not always be true. Emission and excitation spectra can have a Gaussian distribution and optical filters “integrate” the emission spectra over their passband, which may produce nonlinear results. Computational correction of cross talk may be further confounded by low signal-to-noise ratios or high signal levels that saturate the detector. In general, optical mapping of multiple indicators requires the thoughtful design of the excitation band(s), emission filter(s), and possible synchronization of tissue illumination with image acquisition to optimize the signal of each indicator while minimizing optical cross talk (315, 317). For example, precisely timed intervals of excitation light illumination and image acquisition enable single-camera excitation ratiometric optical mapping of APs and/or CaTs (318, 319).

Because of the three-dimensional structure of the heart, optical mapping often requires careful positioning of the camera(s) relative to the tissue to provide a relatively “flat” area to be mapped. This can be done by lightly pressing the tissue against a transparent window, carefully pinning tissue to achieve a flat imaging surface, as often done for atrial tissue (255), or slicing the tissue into thin sections (320, 321). If significant curvature of the tissue persists within the mapped region then wavefronts moving in three dimensions can impact measurements of conduction velocity (169). Technical innovations have enabled multidimensional optical mapping, including panoramic mapping of the entire epicardium (169, 322, 323), simultaneous optical and electrical panoramic mapping of mouse hearts (324), and transmural mapping of thick tissue using transillumination (325) and near-infrared indicators (326, 327).

#### Optical mapping of Langendorff-perfused hearts.

Optical mapping is well-suited for studying excised Langendorff-perfused hearts as optical components can be configured to image APs and/or CaTs at high spatial resolution from any epicardial region (328), including the entire epicardium (329). Hearts are often imaged while perfused and submerged in a crystalloid solution that is pH-buffered using HEPES or sodium bicarbonate (256, 259, 330). Superfusing the heart ensures tight control of temperature and also facilitates the recording of the ECG. Although blood is the most physiologically relevant perfusion fluid and improves the oxygenation and electromechanical function of excised hearts (331), the blood volume of small mammals is often too small to provide enough blood for ex vivo perfusion and the absorption spectrum of hemoglobin interferes with many optical measurements. Perfluorocarbon-based perfusate is an attractive alternative to blood for such studies (258, 332), or blood-perfused tissue can be optically mapped using indicators that emit at near-infrared wavelengths (327, 333).

Commonly used *V*_m_-sensitive indicators include RH237, di-4-ANEPPS, and di-4-ANBDQBS/Di-ANBDQPQ. These may be combined with indicators to measure other parameters, such as intracellular Ca^2+^ (305, 334–336). It is common to load perfused tissue with an exogeneous indicator using a bolus dose, where a high concentration of indicator is slowly injected over several minutes through an injection port located near the cannula (304, 335–337). Another approach is to perfuse the heart with perfusate containing 1–5 µM of indicator for several minutes (247, 338). A less common approach is to incubate the tissue in superfusate containing a high concentration of indicator, while simultaneously perfusing with an indicator-free solution (255). Although this greatly reduces the signal-to-noise ratio, it facilitates selective imaging of the epicardium by minimizing signal contribution from subepicardial tissue (339).

Heart motion during contraction will distort optical recordings by changing the location and intensity of emitted light in relation to the camera. To reduce or eliminate this “motion artifact,” agents known as excitation-contraction uncouplers are frequently used in optical mapping studies to inhibit crossbridge cycling (260, 340), but they may also have metabolic impact (262, 263). We recently suggested best practices for using one of the most common uncoupling agents, blebbistatin (262). As an alternative, motion artifacts can be minimized by gently constraining the heart for short intervals during a recording (334, 338). Recent developments in binocular imaging, tracking algorithms, and computational hardware have enabled optical mapping studies of unrestrained contracting hearts (319, 341, 342).

#### Recording of the optical action potential.

Optical APs are most often recorded from myocardial tissues using a CCD or CMOS camera, or photo-diode array. Each pixel of the detector records bandlimited light emitted from the tissue during membrane depolarization and repolarization that represents the optical AP, the amplitude of which is determined by the amount of indicator present in the membrane, the optical properties of the tissue, and the intensity of the excitation light. These parameters vary across the surface of the tissue and are also different for each experiment. To account for this, optical AP signals are often normalized to have the same range at each pixel, which uniformly scales their amplitude across the mapped region to enable spatiotemporal AP dynamics to be viewed as a movie, however, such normalization is not required for determining activation or repolarization times.

The amplitude of the optical AP also depends on the volume of myocytes imaged by a single pixel, which is determined by the optical magnification, pixel size, light penetration, and light scattering from surrounding tissue. The optical AP from a single cardiomyocyte resembles the AP recorded by patch clamp, with the exception that the optical signal is relative and should be expressed in arbitrary fluorescent units instead of millivolts (343). Notably, the depolarization phase of the AP is slower when recorded optically versus electrically from cardiac tissues (274, 343, 344). A slower optical upstroke in tissue is the result of imaging the volume of cardiomyocytes that are in focus at a pixel, while also acquiring scattered light from neighboring cells that depolarize at different times. Accordingly, the upstroke of the optical AP contains local, as well as remote components, similar to the unipolar electrogram (345). Local activation is considered to occur when the change in fluorescence is at maximum (d*F*/d*t*_max_), which appears earlier in the myocardium imaged close to the pacing site and later in the distal myocardium (345). The use of a high magnification reduces the optical pathlength, which should increase the intensity of the signal, but this marginal increase is typically offset by the fact that fewer myocytes contribute to the emitted light collected at each pixel, resulting in a reduced signal amplitude.

In relatively small hearts, including mice, or during high magnification mapping, the speed of the AP wavefront requires consideration. With very fast total activation times, the sampling frequency must be sufficiently high to capture the activation sequence. During sinus rhythm, the total epicardial activation time of the mouse heart is often <∼6 ms (214, 346). Under sampling, in this case <2 kHz, may result in the inaccurate detection of early epicardial breakthrough during sinus rhythm. For most detectors with more than 50 × 50-pixel sensors, and a magnification that enables imaging of the majority of the anterior surface of a rabbit heart, a sampling rate of <0.5 kHz may be sufficient. However, this may not satisfy the Nyquist criteria for imaging an AP upstroke that only lasts for 1 to 2 ms [although some have estimated it to occur in less than 0.5 ms (347)]. In addition, the dyes themselves transduce *V*_m_ at different rates, so that “fast response” dyes like di-4-ANEPPS are often measured with sampling rates near 1 kHz (348). It should also be noted that CCD and CMOS-based sensors integrate the emission light during the sample time, and therefore the signal amplitude negatively correlates with sampling frequency. Consequently, it may be useful to use a higher sampling frequency when experiments are concerned with rapidly changing parameters (e.g., AP depolarization) compared with more slowly changing parameters (e.g., AP repolarization). To improve the signal-to-noise ratio, spatial binning of pixels or temporal filtering is often required, although it can affect the shape of the AP (349). Temporal signal averaging (e.g., averaging several APs over time) is an alternative approach for reducing noise, but it is best suited for experiments where beat-to-beat variations are minimal, such as occurs during steady-state pacing and therefore could not be applied for arrhythmia analysis.

#### Recording of the optical Ca^2+^ transient.

There are two different functional classes of calcium-sensitive dyes, often referred to as single wavelength or ratiometric dyes. The ratiometric dyes are further classified by whether they have two excitation or two emission peaks in their spectra. The relative amplitudes of the peaks depend on the proportion of intracellular Ca^2+^ ions bound to the fluorophore, making the dissociation constant, or *K*_d_ (the point at which half of the dye molecules are bound to Ca^2+^) an important parameter to consider, which should be matched to the expected Ca^2+^ concentration to be recorded. Given that intracellular Ca^2+^ often rises from ∼200 nM in diastole to approximately >1 μM in systole (350), Ca^2+^-sensitive dyes with a moderate affinity (300–700 *K*_d_) are most frequently used for intracellular Ca^2+^ recording. Ca^2+^-sensitive dyes with a lower *K*_d_ can saturate before the peak of the CaT and can interfere with calcium buffering (351), whereas dyes with a significantly higher *K*_d_ can produce signals with low amplitude and poor signal-to-noise ratios [but may be useful for recording Ca^2+^ dynamics from particular subcellular compartments, e.g., the SR (155)].

As the use of Ca^2+^-sensitive dyes are subject to many of the same issues as *V*_m_-sensitive dyes (e.g., light intensity, dye loading, its degradation, and its washout), a single wavelength signal must be scaled for every pixel and signal intensity in this case reflects relative, not absolute changes in [Ca^2+^]_i_. Dual-wavelength dyes are less affected by some of these artifacts but require a different and more involved calibration method involving the recording of signals at two emission wavelengths (corresponding to peaks that occur at wavelengths higher and lower than the wavelength at which fluorescence intensity changes in opposite direction with the binding of Ca^2+^, the “isosbestic point”) (130).

#### Software packages for optical data analysis.

Optical mapping data can be analyzed using various platforms (e.g., MATLAB, R, or Python). Often, custom-made analysis routines are developed by research groups, some of which have been published and are publicly available [e.g., Rhythm (349), ORCA (119), Electromap (120), Kairosight (312), Cosmas (352)], as well as numerous packages developed by various camera manufacturers. To improve reproducibility, investigators should report not only the software used for analysis, but also any specific settings and the method used to select activation, repolarization, etc., as many of these software packages have multiple options that can be tailored to a specific data set.

## SUMMARY AND FUTURE DIRECTIONS

Cardiac arrhythmias are a primary cause of morbidity and mortality, accounting for 10–20% of all deaths worldwide (353–355). Preclinical animal models have proven to be an invaluable tool for understanding the complex mechanisms that cause arrhythmogenesis, developing novel therapies for the treatment or management of arrhythmias, and testing the efficacy and safety of medications. As highlighted throughout these Guidelines, a variety of experimental techniques can be used to evaluate cardiac electrophysiology at the whole animal, intact heart, or tissue level. A brief summary of these techniques is included in Table 1, which lists common measurements, strengths, and limitations of each approach. Although we have described these technical approaches as individual entities, many of these methodologies can be used sequentially in the same animal (e.g., in vivo ECG recordings followed by ex vivo optical mapping), and many of the described techniques also allow for additional follow-up mechanistic studies (e.g., molecular and histological assessment). Notably, the described techniques can be broadly applied to several different animal species, each of which has distinct advantages and disadvantages depending on the research question being addressed (7, 20, 356). For some studies, a more comprehensive approach may be needed that combines in vivo and ex vivo assessments, isolated cardiomyocyte measurements, and/or computational modeling.

In the presented guidelines, we reviewed current methods and approaches that are most commonly used for measuring cardiac electrophysiology and arrhythmias in small animal models, although recent advances in the field are likely to enhance and evolve these technical approaches in the near future. For example, contactless “all optical” cardiac electrophysiology techniques use genetically encoded voltage and/or calcium indicators (for optical mapping) in combination with optogenetic actuation to precisely control cardiac excitation via light-sensitive ion channels (357). All-optical cardiac electrophysiology has been successfully used in vitro using hiPSC-CMs for preclinical drug testing (358, 359), assessment of neuron-cardiomyocyte interactions (360), and modulation of cardiac electrical activity in 2-D/3-D hiPSC-CM preparations (361) and intact, isolated hearts (362, 363). Advancements in microendoscopy (364) and fabrication of miniaturized LED devices (365) can be used to optimize light delivery in vivo, and one day, these tools may be used as optical alternatives to electrical devices [e.g., pacemakers, defibrillators (366, 367)]. Miniaturized, flexible, and stretchable electronics can be used for implantable optogenetic devices (e.g., LEDs), and these conformable devices can also be used to modulate and/or monitor biochemical and biophysical signals. As an example, 3-D multifunctional integumentary membranes (3-D-MIMs) have been engineered using elastic membranes that conform to the shape of the heart akin to the pericardium, and provide electrical stimulation for optical mapping, strain gauges, and ECG/pH/temperature sensors (365). These multiparametric devices can provide information on the interactions between metabolism, electrical, and mechanical activity. Collectively, these emerging technologies and approaches will no doubt expand our ability to comprehensively assess and interrogate cardiac electrophysiology and arrhythmia mechanisms in animal models.

## GRANTS

This work was supported by MAESTRIA Grant 965286 and British Heart Foundation Accelerator Award AA/18/2/34218, DZHK (to L.F.); National Institutes of Health (NIH) Grants R01HL136389, R01HL163277, and R01HL147108 (to N.L); NIH Grants R01HL139472 and R01HD108839 (to N.G.P.); NIH Grants R01HL153042 and R01HL141343 (to B.P.D.); Canadian Institutes of Health Research (CIHR) Grant MOP 342562, Natural Sciences and Engineering Research Council of Canada Grant RGPIN-2022-03150, Government of Canada’s New Frontiers in Research Fund NFRFE-2021-00219, and Heart and Stroke Foundation of Canada Grant G-22-0032127 (to T.A.Q.); CIHR Grants PJT166105 and PJT180474 and Heart and Stroke Foundation of Canada Grant G-22-0032033 (to R.A.R.); NIH Grants R01HL141214, R01HL139738, and R01HL146652 (to A.V.G.); NIH Grants R01HL156652 and R01HL135096 (to T.J.H.); NIH Grants R01HL146169, R01HL147279, and R01HL144157 (to M.W.K.); NIH Grant R01HL133127 and American Heart Association Basic Project Grant 18SFRN34230125 (to K.M.); Netherlands CardioVascular Research Initiative CVON (Dutch Heart Foundation, Dutch Federation of University Medical Centres, ZonMw, and Royal Netherlands Academy of Sciences) Grants CVON2018-30 PREDICT2 and CVON2015-12 eDETECT (to C.A.R.); NIH Grant R35HL144980 (to B.C.K.); NIH Grants R01HL138003, R01HL141855, and R01HL102298 (to S.P.); NIH Grants R01HL085727, R01HL085844, and R01HL137228 and VA Merit Review Grants I01 BX000576 and I01 CX001490 (to N.C.); NIH Grants R01HL130212 and R01HL163274 and Burroughs Wellcome Fund CAMS (1009884) (to S.L.R.); and NIH Grants R01HL111600 and OT2OD026580 (to C.M.R.).

## DISCLOSURES

L.F. has received institutional research grants and nonfinancial support from European Union, DFG, British Heart Foundation, Medical Research Council (UK), NIHR, and several biomedical companies. L.F. is listed as an inventor of two patents (Atrial Fibrillation Therapy WO 2015140571, Markers for Atrial Fibrillation WO 2016012783). None of the other authors has any conflicts of interest, financial or otherwise, to disclose.

Crystal Ripplinger is an editor of *American Journal of Physiology-Heart and Circulatory Physiology* and was not involved and did not have access to information regarding the peer-review process or final disposition of this article. An alternate editor oversaw the peer-review and decision-making process for this article.

## AUTHOR CONTRIBUTIONS

C.M.R. and N.G.P. conceived and designed research; C.M.R., A.V.G., M.W.K., N.L., and N.G.P., prepared figures; C.M.R., A.V.G., M.W.K., B.J.B., N.C., B.P.D., L.F., T.J.H., B.C.K. N.L., K.T.M., S.P., T.A.Q., C.A.R., S.L.R., R.A.R., and N.G.P., drafted manuscript; C.M.R., A.V.G., M.W.K., B.J.B., N.C., B.P.D., L.F., T.J.H., B.C.K., N.L., K.T.M., S.P., T.A.Q., C.A.R., S.L.R., R.A.R., and N.G.P., edited and revised manuscript; C.M.R., A.V.G., M.W.K., B.J.B., N.C., B.P.D., L.F., T.J.H., B.C.K., N.L., K.T.M., S.P., T.A.Q., C.A.R., S.L.R., R.A.R., and N.G.P., approved final version of manuscript.
